# Brain functional and structural magnetic resonance imaging of obesity and weight loss interventions

**DOI:** 10.1038/s41380-023-02025-y

**Published:** 2023-03-14

**Authors:** Guanya Li, Yang Hu, Wenchao Zhang, Jia Wang, Weibin Ji, Peter Manza, Nora D. Volkow, Yi Zhang, Gene-Jack Wang

**Affiliations:** 1 Center for Brain Imaging, School of Life Science and Technology, Xidian University & Engineering Research Center of Molecular and Neuro Imaging, Ministry of Education, Xi’an, Shaanxi 710071 China; 2 International Joint Research Center for Advanced Medical Imaging and Intelligent Diagnosis and Treatment & Xi’an Key Laboratory of Intelligent Sensing and Regulation of trans-Scale Life Information, School of Life Science and Technology, Xidian University, Xi’an, Shaanxi 710126 China; 3 Laboratory of Neuroimaging, National Institute on Alcohol Abuse and Alcoholism, Bethesda, MD 20892 USA

**Keywords:** Neuroscience, Addiction, Diagnostic markers, Biotechnology

## Abstract

Obesity has tripled over the past 40 years to become a major public health issue, as it is linked with increased mortality and elevated risk for various physical and neuropsychiatric illnesses. Accumulating evidence from neuroimaging studies suggests that obesity negatively affects brain function and structure, especially within fronto-mesolimbic circuitry. Obese individuals show abnormal neural responses to food cues, taste and smell, resting-state activity and functional connectivity, and cognitive tasks including decision-making, inhibitory-control, learning/memory, and attention. In addition, obesity is associated with altered cortical morphometry, a lowered gray/white matter volume, and impaired white matter integrity. Various interventions and treatments including bariatric surgery, the most effective treatment for obesity in clinical practice, as well as dietary, exercise, pharmacological, and neuromodulation interventions such as transcranial direct current stimulation, transcranial magnetic stimulation and neurofeedback have been employed and achieved promising outcomes. These interventions and treatments appear to normalize hyper- and hypoactivations of brain regions involved with reward processing, food-intake control, and cognitive function, and also promote recovery of brain structural abnormalities. This paper provides a comprehensive literature review of the recent neuroimaging advances on the underlying neural mechanisms of both obesity and interventions, in the hope of guiding development of novel and effective treatments.

## Introduction

Global prevalence of obesity has increased substantially over the past 40 years, from less than 1% in 1975, to 6–8% in 2016 [1]. In 2016, more than a third of adults worldwide were classified as overweight or obese, as were 41 million children under the age of five [2]. Data from 2017 to 2018 indicate that more than 42.4% of American adults are living with obesity, an increase from 30.5% in 1999–2000 [3]. The prevalence of obesity in Chinese adults increased from 3.1% with mean BMI of 22.7 kg/m^2^ in 2004 to 8.1% with mean BMI of 24.4 kg/m^2^ in 2018 [4], and about half of adults and a fifth of children have overweight or obesity [5]. China has overtaken the US as the most obese nation, with almost 90 million obese people, and the US is close behind with over 87 million [6]. Obesity affects people of all ages and demographic backgrounds [7] and increases risk to a range of diseases, including type 2 diabetes, cardiovascular disease, and cancer, and is considered a risk factor for dementias including Alzheimer’s disease [8–10]. Thus, improved understanding of the psychophysiological mechanisms that regulate appetite and weight are essential for the development of effective treatments to combat obesity.

Over the past two decades, neuroimaging particularly magnetic resonance imaging (MRI), including functional (fMRI), structural (sMRI) and diffusion tensor imaging (DTI) has become a popular and rapidly advancing tool for investigating the neurobiology underlying variation in eating behavior related to obesity in humans. fMRI infers local neuronal activity from blood-oxygen-level dependent (BOLD) changes in the paramagnetic properties of hemoglobin [11]. fMRI study paradigms examine the brain’s response to visual, olfactory, or gustatory (taste) food vs. control cues, or to different categories of food cue (i.e., high- vs. low-palatability, high- vs. low-calorie), or to different states (i.e., pre- vs. postprandial, hunger vs. satiety, fasting vs. post-meal)[12–31]. Besides these cue reactivity studies, growing body of evidence highlights obesity-associated cognitive dysfunctions including impaired decision-making [32], inhibitory control [33], learning/memory [34] and attention [35]. To test the impact of obesity on these cognitive functions, researchers have used a number of experimental designs, including willingness to pay [36–39], delay discounting [32, 40–43], learning [34], episodic memory [44], food Stroop [35] and Go-No/Go [33] tasks. Resting-state fMRI (RS-fMRI) is also utilized to assess resting-state brain activity before and after ingestion of a substantial calorie food or weight loss intervention [45–63]. sMRI is frequently used to obtain anatomical information, including gray-(GM) and white-matter (WM) volumes [64–80], and cortical morphometry [81–85]. DTI is a highly sensitive tool for assessing the integrity of WM tracts as quantified by fractional anisotropy (FA), mean-(MD), axial-(AD), and radial diffusivity (RD) [86–96]. In addition, multi-modal MRI is an emerging tool to examine brain functional and structural changes simultaneously [55, 97–117].

**Fig. 1:**
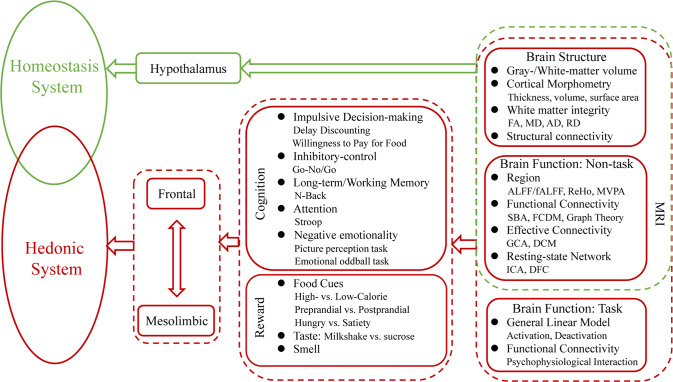
MRI studies show the association between obesity and abnormal function in brain regions and circuitry associated with homeostasis and hedonic process. FA fractional anisotropy, MD mean diffusivity, AD axial diffusivity; RD radial diffusivity, ALFF amplitude of low-frequency fluctuation; ReHo, regional homogeneity, MVPA multi-variate pattern analysis, SBA seed-based analysis, FCDM functional connectivity density mapping, GCA Granger causality analysis, DCM dynamic causal model, ICA independent component analysis, DFC dynamic functional connectivity.

These aforementioned studies have shown associations between obesity and abnormal function in brain regions and circuitry associated with homeostasis [118] as well as hedonic processes associated with reward/motivation [12, 13, 16, 18, 21, 25], emotional reactivity [16, 19] and inhibitory control [16, 25, 30]. Alteration in consumption of food based on energy balance forms the foundation of the homeostatic control of appetite: following a meal, appetite is suppressed, whereas, following significant energy expenditure, hunger is boosted. These hunger and satiety signals are regulated by changes in circulating concentrations of nutrients and orexigenic/anorectic gut hormones and peptides [119]. The hypothalamus is widely recognized as the gatekeeper for this processing task, and there is mounting evidence that hypothalamic dysfunction is implicated in the pathogenesis of obesity [118]. Hedonic functions are processed mainly by frontal-mesolimbic regions including the frontal cortex (dorsal lateral prefrontal cortex-DLPFC, anterior cingulate cortex-ACC, orbitofrontal cortex-OFC) [16, 25], striatum (nucleus accumbens-NAc, caudate, putamen) [12, 13, 18, 30], limbic regions (insula, amygdala-AMY, hippocampus-HIPP) and thalamus [16, 19]. Homeostatic and hedonic systems primarily participate in the control of appetite and food intake regulation, and there is extensive cross-modulation between them [120]. Imbalance or dysregulation between them may result in eating disorders [120] (Fig. 1).

A variety of prevention efforts and interventions have been developed to address the obesity epidemic. Traditional dietary and lifestyle modifications (i.e., physical exercise) offer the mainstays of obesity intervention [40, 121], and anti-obesity drugs may be taken in conjunction to reduce appetite or fat absorption [122–125]. Bariatric surgery, including Roux-en-Y gastric bypass (RYGB) and laparoscopic sleeve gastrectomy (LSG)[126], has emerged as the most effective clinical treatment for morbid obesity, which can not only cause profound changes in appetite-regulating peptides and neuroendocrine function [127] but also promote recovery of obesity-associated brain functional and structural abnormalities [128–131]. In addition, non-invasive neuromodulation techniques including transcranial direct current stimulation (tDCS), transcranial magnetic stimulation (TMS), and neuromodulation/neurofeedback have been introduced as potential strategies to ameliorate brain dysfunction, improve eating behaviors and weight loss [132–134].

We provide an updated review of recent advances (2016–2022) falling into the scope of MRI studies on eating behavior related to obesity and weight loss interventions. We gathered relevant literature from Scopus and PubMed published between Jan 2016 and July 2022, using the following search terms in different combinations: ‘obesity’, ‘brain’, ‘structure’, ‘function’, ‘bariatric surgery’, ‘neuroimaging’, ‘RYGB’ and ‘sleeve gastrectomy’. Articles written in English were screened for relevance. We identified accumulating evidence for prominent associations between obesity and abnormal function in brain circuits associated with homeostatic maintenance as well as hedonic processes involved with reward/motivation, emotional reactivity, and inhibitory control. These complicated processes may be partly explained by the well-established theoretical models such as the “competing neural systems model”, which describes how food-related reward-seeking overpowers inhibitory systems mediating the control of food intake, leading to suboptimal decision-making characterized by impulsive choices. The current review paper focuses on reward and cognitive control circuitries based on that specific model of obesity. We start with the impact of obesity on brain function and structure: for brain dysfunction, neural response to food cues, taste and smell, resting-state activity and functional connectivity, and cognitive functions including impulsive decision making, inhibitory control, learning/memory, and attention will be discussed; for brain structure, this review will focus on GM/WM volume and WM integrity reductions as well as decreases in structural connectivity. Then, anti-obesity interventions will be introduced, and the underlying neural mechanisms of how these treatments regulate brain dysfunction to improve eating behavior will be illustrated. Finally, we propose possible future studies to better unravel the underlying neural mechanisms of obesity and of its therapeutic interventions, and to help develop novel and effective treatments.

## MRI studies related to obesity

### Neural responsive studies

#### Food cues

Food cue reactivity task is a well-established experimental design to test neural responses to food cues between obese and normal-weight individuals. Compared to normal-weight participants, in responses to food vs. non-food, or high- vs. low-calorie food cues obese individuals showed greater brain activations in reward processing regions including caudate, putamen, nucleus accumbens (NAc), OFC, ACC, ventral tegmental area (VTA) and somatosensory cortex [12, 13, 18, 135–137], in regions involved with emotional regulation such as insula, amygdala, thalamus and pregenual ACC (pgACC) [16, 19], in regions implicated in memory, imagery and executive functions including DLPFC, superior frontal gyrus (SFG), fusiform gyrus (FFA) and parietal lobe [19] and in self-referential processing such as precuneus and posterior cingulate cortex (PCC) [14, 30]. In contrast, obese individuals showed lower activation in frontal cortical [16, 30] and temporal regions involved in cognitive control [25], and in the dorsal ACC (dACC) and head of caudate implicated in attentional and salience processing [16, 21].

Together, these studies suggest that obesity is consistently associated with heightened or abnormal responses to visual food cues in a distributed network of brain regions involved in reward/motivation and emotion/memory and with reduced activation in areas associated with cognitive-control/attention. Although it is not possible to infer the precise cognitive functions underlying these brain activation group differences, this could reflect difficulty to inhibit craving upon food cue stimulation in individuals with obesity.

#### Taste/olfactory cues

Brain responses to the taste and smell of food also are different between obese and normal weight participants. Compared to lean counterparts, obese individuals in response to glucose/fructose/milkshake vs. tasteless solution showed greater activation in premotor areas, superior parietal lobule (SPL) and visual cortex belonging to the dorsal attention network, in medial prefrontal cortex (MPFC), supplementary motor area (SMA), precuneus/PCC, middle temporal gyrus (MTG), striatum, gustatory area (anterior insula and frontal operculum) and somatosensory cortex comprising the mouth area, and enhanced amygdala-ventromedial prefrontal cortex (VMPFC) connectivity; whereas they showed decreased activation in AMY, HIPP, hypothalamus, which are involved with feeding regulation and in VMPFC and ventrolateral prefrontal cortex (VLPFC) [15, 20, 22, 27, 31, 138, 139]. In addition, obese relative to normal weight participants showed greater responses to odors of high vs. low energy dense food or hunger vs. satiety state in the IFG, cerebellar vermis, anterior insula, putamen, MTG, primary olfactory and odor memory areas; and lower response in reward (i.e., caudate, lentiform nucleus), frontal and sensory areas [23, 24]. These findings are interpreted to reflect abnormal brain responses in obese individuals to taste and olfactory cues in regions involved with decision making, response inhibition, reward, olfactory and memory processing, that might underlie eating behaviors that promote excessive food consumption and weight gain.

#### Cognition tasks

Obese individuals have higher sensitivity to food cues, as evidenced by heightened brain reward circuitry activity during the encoding of sensory stimuli. During food picture viewing and food choice tasks, obese participants also exhibit greater activation in frontal regions implicated in self-regulation and control of eating behaviors, suggesting stronger engagement of executive-control to suppress food reinforcers (reviewed in [140]). Thus, neurocognitive tasks, including delay discounting, willingness to pay, Go/No-Go, Stroop and learning/memory, have been adopted to measure different domains of executive functions including impulsive decision making, inhibitory control, inattention, and learning/memory [140]. Impulsive decision-making refers to the tendency not to delay gratification and to prefer immediately available rewards. It is typically tested with willingness to pay for food/monetary incentive delay tasks and the delay discounting task, which requires participants to make a series of choices between smaller immediate reward and larger delayed rewards [32, 36–39, 41–43, 141]. It is measured by the discounting rate, which represents how quickly the subjective value of a reward decreases as a function of delayed time and choice behavior [43]. During reward evaluation (food/monetary), obese participants showed greater activations in the striatum, insula, AMY, OFC, VMPFC, and lower functional connectivity of frontal-striatal/ventral striatum-insula circuitries, relative to controls [36–38, 41]. During decision-making, obese individuals showed increased activation in DLPFC, ventral PCC, angular gyrus, inferior-(IPG) and posterior parietal gyrus, and decreased activity in the insula and functional connectivity of DLPFC-IPG/angular gyrus-caudate [32, 39, 42, 43, 141]. These findings might reflect that obese participants prefer the reward of immediate food intake when exposed to external food cues, rather than the delayed reward of health brought about through diet management and physical activity. Thus, sensitization to food cues might support the possibility of a food reward overriding cognitive-control and resulting in dysfunctional reward-based decision-making, ultimately contributing to impulsive maladaptive eating behaviors [43, 142, 143].

Inhibitory control is associated with the ability to suppress prepotent motor responses [140]. Go/No-Go is a standard task to test inhibition, in which individuals are asked to answer as quickly as possible when a repeated visual stimulus appears (Go signal) but to inhibit their response when a rare stop signal appears (No-Go signal). Relative to controls, obese females showed greater activation in the insula, caudate, putamen and precuneus, and lower activation in the Rolandic operculum and thalamus during response inhibition [33]. Attention is related to the ability to focus on specific activities while suppressing the response to distracting stimuli [140]. The Stroop task is typically used to measure the inattention domain of impulsivity, which requires participants to identify the color of a written color word, without reading the word itself. When the word is printed in a color that is incongruent with the word, there is a conflict between word reading and color naming [140]. One study using a food and emotion word Stroop task showed that greater obesity scores (an aggregate measure of body mass index (BMI), waist circumference and waist-to-hip ratio) were associated with lower lateral PFC responses during food attentional bias [35].

With regards to learning and memory, probabilistic learning task can characterize the neural underpinnings of reinforcement-based learning. Obese patients made a significantly lower number of correct choices and earned less money, and had greater activation in MPFC and functional connectivity of ventral striatum-insula during outcome learning, relative to controls [34]. Further, obesity may be associated with episodic memory deficits [144]. Cheke et al. adopted the What-Where-When episodic memory test to assess the ability of remembering integrated item, spatial, and temporal details of previously encoded complex events, and though they did not observe behavioral differences between lean and obese participants, results indicated that obesity is associated with functional changes in brain areas involved with memory (i.e., HIPP, angular gyrus and DLPFC) [44].

Overall, current evidence from neurocognitive tasks suggests that obese individuals generally show greater impulsive decision-making and attentional bias in response to food cues. In addition, growing evidence showed differences in fMRI activity during cognitive control tasks in obesity relative to normal weight control group.

### Resting-state fMRI studies

Resting-state fMRI (RS-fMRI) has been employed to investigate alterations in regional activity and/or resting-state functional connectivity (RSFC) integrity of resting-state networks (RSNs) related to food-intake in obese participants. Obese compared to normal weight participants showed greater regional RSFC of insula/operculum-cuneus, MTG-OFC, DLPFC-visual cortex, NAc-MPFC, caudate-somatosensory cortex, caudate-ITG/STG/FFA/AMY/HIPP, globus pallidus (GP)-putamen, VMPFC-ITG/SMA/FFA/insula/Postcentral gyrus; and lower regional RSFC of insula-dACC, MTG-PCC/precuneus/angular gyrus, caudate-precuneus, PFC-striatum, which were associated with eating behaviors and BMI [45, 47, 48, 50, 53–55, 59, 62, 63]. Obese subjects also showed reduced FC strength in the VMPFC and PCC/precuneus within the default-mode network (DMN), dACC within the salience network (SN), bilateral DLPFC-angular gyrus within the frontoparietal network (FPN) which correlated with disinhibition and BMI; and increased FC between SN and FPN driven by altered FC of INS/ACC-angular gyrus [49, 56]. Functional network connectivity analysis showed increased FC between SN and emotional regulation network, between BG and FPN/DMN/SN/executive-control network (ECN); and decreased FC between DMN and FPN/ECN [51, 57, 58, 145]. In addition, graph theory analyses revealed obese subjects exhibited reduced nodal degree/efficiency in frontal (OFC, rACC), striatal (caudate/putamen/NAc, pallidum) and limbic regions (insula, AMY, HIPP/PHIPP) and thalamus, as well as decreased connectivity of cortico-striatal/cortico-thalamic network and subnetwork associated with the right ACC [46, 52, 61]. Thus, obese individuals have greater RSFC between regions involved in metabolic sensing/interoception and regions involved in reward processing, and lower RSFC in regions involved in interoceptive processing/cognitive control, suggesting abnormal communication between multiple brain circuits in obese patients at rest.

RS-fMRI was also employed to assess brain activity before and after a meal and findings were similar as those reported above [146–148]. In order to further dig into why obese participants show heightened activation of regions involved with reward processing, recent studies combined RS-fMRI with a food cue reactivity task observed overlapping activated regions in limbic circuits including HIPP and AMY [101, 111], and BMI-related activation within DMN both when exposed to food cues and at rest [106], suggesting that their regional responses to high-calorie food cues were mediated by their resting-state activity.

### Structural MRI studies

#### T1 (GM/WM volume, morphometry)

Many studies have shown that obesity has a significant negative impact on GM and WM integrity, and BMI is negatively associated with GM/WM volumes and cortical morphometry. sMRI has been employed to characterize obesity-associated brain structural alterations. Cortical morphometric analysis indicates that obese individuals compared to controls showed lower cortical thickness in frontal regions including VMPFC, SFG, IFG and OFC, which are regions associated with executive function, and temporal and occipital cortices which are associated with visceral fat level and food addiction symptom severity, and in the somatosensory cortex [76, 81–85]. Obese participants also showed lower GM volumes in frontal (i.e., MPFC, OFC) and mesolimbic regions (i.e., ACC, caudate, putamen, NAc, insula, AMY, HIPP, pallidum), thalamus, anterior portion of the corpus callosum (CC) [64, 66, 67, 71–77, 79, 80, 103, 115], which were negatively associated with BMI. Baseline brain structure was able to predict weight loss following a weight reduction intervention [70].

#### DTI

Growing evidence showed that obesity not only influences integrity of GM but also WM and structural connectivity. Obese participants compared to controls have lower FA/AD in the anterior and posterior thalamic radiation, the inferior fronto-occipital fasciculus, inferior-(ILF) and superior longitudinal fasciculus (SLF), CC, uncinate fasciculus, internal and external capsule, corticospinal tract and the cingulum (cingulate gyrus and HIPP), anterior corona radiata [88–91, 95, 96], and lower MD in the right globus pallidus and right putamen [94]; and greater AD/MD in the forceps minor, anterior thalamic radiation, superior and inferior longitudinal fasciculus [87, 93]. These fiber tract alterations link limbic structures with prefrontal regions associated with abnormal reward processing and cognitive performance.

A limitation for these cross-sectional reports in obese adults is that they cannot differentiate if the brain structural and functional differences precede or follow obesity. In this respect prospective, sMRI studies have allowed to ascertain brain changes that emerge as BMI increases [149–151]. Specifically, Shaw et al. reported that increases in BMI at midlife (44–49 yrs) was associated with increased cortical thinning in PCC and reduced cortical thickness in right supramarginal and frontal cortices, whereas decreases in BMI was associated with increased cortical thinning in the right caudal middle frontal cortex [149]. Franz and colleagues revealed that obese relative to normal weight individuals had thinner cortex in multiple frontal and temporal lobe regions [150]. A five-years longitudinal sMRI study showed that higher baseline BMI was associated with greater decline in temporal and occipital GM volumes, and changes in BMI over the 5-year period was associated with change in HIPP volume [151]. Another 9-year follow-up study showed baseline waist circumference was associated with decreasing HIPP volume, particularly in men, and increasing WM hyperintensity volume in women and men [152]. These findings provide evidence that obesity causes reductions in GM and WM.

### Multi-modal MRI studies

Besides those uni-modal MRI reports, a growing number of studies have adopted multi-modal MRI to examine obesity-related brain abnormalities. One study employing a spatial/verbal working memory task with fMRI and sMRI showed that higher BMI was associated with greater SN functional connectivity and lower WM volume throughout DMN, ECN and SN than controls [107]. Another similar study showed that BMI negatively correlated with FA in the left ILF/SLF, which together with IQ mediated the relationship between BMI and verbal working memory performance [99]. Other studies used different combinations of cognitive tasks/resting-state with sMRI and DTI [98, 102, 153], or cue reactivity/taste/cognitive tasks with sMRI [104, 105, 108, 113, 116], or sMRI with DTI [97, 100, 109, 117, 154, 155], to investigate brain functional and structural alterations simultaneously. These efforts support the notion that structural changes in brain regions play a prominent role in the functional coupling between those regions, ultimately leading to changes in behavior [156].

Here we attempt to provide a comprehensive literature review of the recent neuroimaging advances on the underlying neural mechanisms of obesity, but note that this is not a systematic review and we have not included all possible studies to date. We primarily focused on evidence congruent with the ‘competing neural systems model’, but crucially, not all studies neatly align with this framework. For example, Doornweerd and colleagues investigated brain responses to visual and taste stimuli in 16 female monozygotic twin pairs, and results showed no statistically significant differences in regional activations when comparing leaner and heavier co-twins [157]. Carbine and colleague tested how N2 and P3 amplitude, event-related potential components that reflect inhibitory control, and fMRI activity in brain regions associated with inhibitory control differed toward high- and low-calorie food stimuli across BMI status, and results showed no main effects or interactions involving BMI or method [158]. There are many reasons contributing to the inconsistent results including differences in participant demographics, experimental design, and data analytic strategies, as well as differences in statistical power to identify significant effects. Thus, future reviews could adopt more strict inclusion criteria to evaluate literature consistencies more systematically.

## Molecular genetics of obesity

Obesity is a multi-factorial disorder. The hypothalamus and its signaling molecules play a critical role in coordinating energy balance and homeostasis, and genetic factors play a crucial role in determining an individual’s predisposition to weight gain and obesity.

### Signaling molecules and regulation of eating behavior

A number of peripheral hormones and peptides participate in central nervous system (CNS) control of appetite and food intake. Palatable foods activate the mesolimbic dopamine (DA) reward system essential for feeding behavior regulation (reviewed in 159). Hunger and satiety signals from adipose tissue (leptin)[159], the pancreas (insulin)[160], and the gastrointestinal tract (cholecystokinin (CCK), glucagon-like peptide-1 (GLP-1), peptide YY3-36 (PYY3-36), and ghrelin)[161] constitute key components in the gut-brain axis-mediated control of appetite, energy expenditure, and obesity. Leptin and insulin are considered long-term regulators of energy balance, whereas ghrelin, CCK, peptide YY, and GLP-1 are sensors related to meal initiation and termination and hence affect appetite and body weight more acutely. These hormones and peptides alter appetite and eating behaviors by acting on hypothalamic and brainstem nuclei, and may directly or indirectly interact with the midbrain DA pathways to impact feeding [161].

### Brain imaging and genetics of obesity

The genetics of obesity are distinguished as syndromic and non-syndromic, and are associated with distinct genetic and clinical consequences (reviewed in 159). Genetic syndromes associated with obesity can present with or without developmental delay [162]. Prader-Willi syndrome, fragile X syndrome, SIM1 deficiency, Bardet-Biedl syndrome, Cohen syndrome, and Albright’s Hereditary Osteodystrophy (AHO) syndrome are associated with obesity with developmental delay. In addition, monogenic obesity including Alstrom syndrome, congenital leptin deficiency, leptin receptor deficiency [163], POMC deficiency [164], PC1 deficiency [165], MC4R deficiency [166], SH2B1 deficiency and variants in the FTO gene [167], and polygenic obesity including β-adrenergic receptor family gene [168], uncoupling proteins gene [169], SLC6A14 gene [170] are associated with obesity without developmental delay [162, 171]. Identification of the genetic causes of obesity is clinically important for genetic counselling and for helping to guide therapeutic interventions [172, 173]. However, further research is needed to link genes implicated in obesity with brain functional and structural changes observed in obesity [77, 174].

## Neuromechanisms of obesity

Functional and structural MRI studies have provided evidence for an imbalance between neural circuits that motivate behaviors (because of their involvement in reward and conditioning) and the circuits that control and inhibit prepotent responses in overeating cases. These circuits involve reward, motivation, learning/memory, and executive control. In vulnerable individuals, consumption of palatable foods in large quantities may disturb the normal balanced interaction among these circuits, resulting in an enhanced reinforcing value of foods and a weakening of inhibitory control. Prolonged exposure to high-calorie diets may also directly alter conditioned learning and therefore reset reward threshold in at-risk individuals. Ultimately, changes in cortical top-down networks that regulate prepotent responses lead to impulsivity and compulsive food intake.

### Reward, conditioning, and motivation

Certain foods, particularly those rich in sugars and fat, are also potently rewarding and can trigger addictive like behaviors in humans [175]. Indeed, high-calorie foods can promote overeating (i.e., actually eating that is not energetic needs) and trigger learned association between the stimuli and the reward (conditioning). Thus, palatable foods represent a powerful environmental trigger, and have the potential to facilitate or exacerbate the establishment of uncontrolled behaviors. In support of this, individuals with obesity show sensitized responses to conditioned cues predictive of food reward. Compared with normal-weight individuals, obese subjects observing pictures of high-calorie food (stimuli to which they are conditioned) showed increased activation in regions of the reward and motivation circuits [176]. Ventral and medial prefrontal regions (including OFC and ventral ACC) become activated with exposure to craving-inducing stimuli. The OFC is similarly involved in attributing salience value to food, helping to assess its expected pleasantness and palatability as a function of its context. There is evidence that the OFC also supports conditioned cue-elicited feeding and that it contributes to overeating, irrespective of hunger signals. Several lines of research support a functional link between OFC impairment and disordered eating, including the reported association between disinhibited eating in obese adolescents and reduced OFC volume [176].

### Learning/memory

Memories can produce an intense desire for food. Multiple memory systems have been proposed including conditioned incentive learning (mediated in part by the NAc and AMY), habit learning (mediated in part by the caudate and putamen), and declarative memory (mediated in part by the HIPP). Conditioned incentive learning about neural stimuli or exaggerated stimulation by overeating generates reinforcing properties and motivational salience even in the absence of food. Through habit learning, well-learned sequences of behaviors are elicited automatically in response to appropriate stimuli. Declarative memory is more about the learning of affective states in relationship to food intake. HIPP function has been implicated in memories of foods or the rewarding consequences of eating in humans. If this function is disturbed, retrieval of memories and environmental cues may evoke more powerful appetite responses essential to obtaining and consuming foods.

### Executive control

The brain top-down control system constitutes a network of frontal regions involved in executive control, goal-directed behavior, and response inhibition. The DLPFC and IFG are components of the system that significantly activated during an individual’s conscious effort to suppress the desire to consume palatable but unhealthy foods. Such DLPFC and IFG activities function to inhibit the desire to consume food, as evidenced by greater cortical activation in those areas that correlate with better self-control in choosing between healthy and unhealthy foods. Vulnerabilities in reward sensitivity, habit, and inhibitory control appear to interact to produce prolonged hyperphagia of highly palatable foods leading to the development and maintenance of obesity. By extension, lower activation of prefrontal and parietal brain regions implicated in inhibitory control, may lead to greater sensitivity to the rewarding effects of highly palatable foods and greater susceptibility to the pervasive temptation of appetizing foods in our environment. This in turn increases overeating in the absence of meeting homeostatic energy needs. Collectively, inhibitory control of food consumption seems to rely on the ability of the brain’s top-down control systems to modulate the subjective valuation of food. Individual differences in food intake regulation may result from structural differences of the DLPFC and/or connectivity with brain valuation regions.

**Fig. 2:**
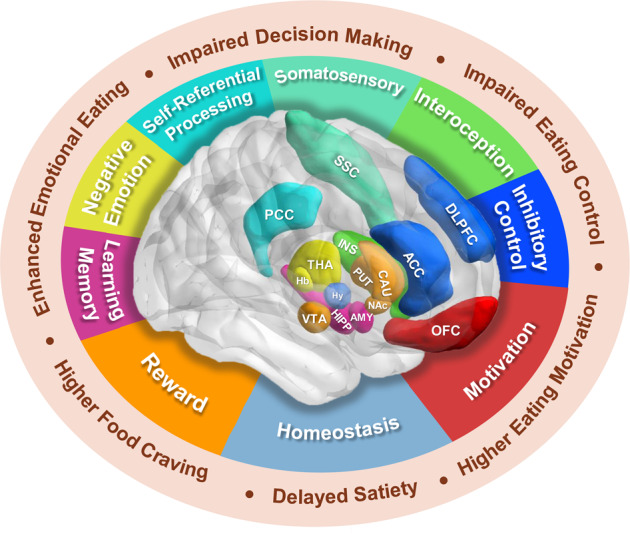
Obesity is associated with abnormality of fronto-mesolimbic circuitry and impaired cognitive functions. Studies have shown associations between obesity and abnormal function in brain regions and circuitry associated with homeostasis as well as hedonic processes associated with reward/motivation, emotional reactivity and inhibitory control. Hedonic functions are processed mainly by frontal-mesolimbic regions including the frontal cortex (DLPFC, ACC, OFC), striatum (NAc, CAU, PUT), limbic regions (INS, AMY, HIPP), and THA. Homeostatic and hedonic systems primarily participate in the control of appetite and food intake regulation, and there is extensive cross-modulation between them. Imbalance or dysregulation between them may result in eating disorders. DLPFC dorsal lateral prefrontal cortex, ACC anterior cingulate cortex, OFC orbitofrontal cortex, NAc nucleus accumbens, CAU caudate, PUT putamen, INS insula, AMY amygdala, HIPP hippocampus, THA thalamus, Hy hypothalamus, VTA ventral tegmental area, Hb habenula, PCC posterior cingulate cortex, SSC somatosensory cortex.

Brain imaging with fMRI, sMRI and DTI expanded our understanding of the neural mechanisms underlying human obesity, and in general have reported consistent findings of functional and structural changes in brain regions and circuitry associated with homeostasis (i.e., hypothalamus) and with hedonic processes. Most implicate changes in the meso-striatal-cortico-limbic circuitry including DLPFC, OFC, ACC, CAU, putamen, NAc, VTA, INS, AMY, and HIPP as well as in thalamus, habenular, PCC and somatosensory cortex (Fig. 2, Table 1, Suppl Table 1). These brain alterations are likely to reflect not only the effects of the dysregulation of orexigenic and anoroxigenic peripheral hormones and neuropeptides [177] but also the effects of genetics and chronic low-grade inflammation associated with obesity [178]. It is also possible that in some instances some of the functional and structural changes preceded the obesity phenotype but increased the risk for overeating and obesity. Longitudinal studies are needed to pre-existing changes in brain function and structure in frontal, striatal, and limbic regions increase risk for overeating and obesity.

**Table 1:**
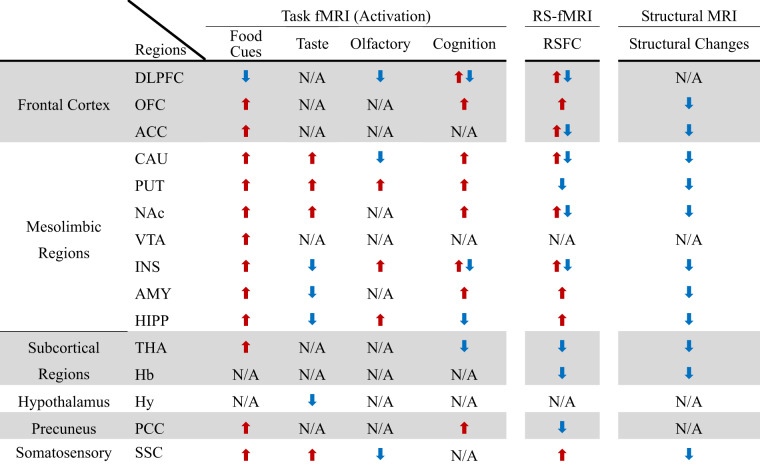
Functional and structural changes in brain regions in people with obesity.

*DLPFC* dorsolateral prefrontal cortex, *OFC* orbitofrontal cortex, *ACC* anterior cingulate cortex, *CAU* caudate, *PUT* putamen, *NAc* nucleus accumbens, *VTA* ventral tegmental area, *INS* insula, *AMY* amygdala, *HIPP* hippocampus, *THA* thalamus, *Hb* habenula, *PCC* posterior cingulate cortex, *Hy* hypothalamus, *SSC* somatosensory cortex. *fMRI* functional magnetic resonance imaging, *RS-fMRI* resting-state fMRI, *RSFC* resting-state functional connectivity, *N/A* Not Applicable, red arrow represents the increase, blue arrow depicts the decrease.

## MRI studies related to weight loss treatments

Comparing currently obese and lean people gives us useful information about the neurobiology of obesity, but does not allow us to infer whether neurological abnormalities precede, follow, or simply accompany the obese state. Examining relationships between brain activation and weight change longitudinally may help illuminate temporal order, and therefore causal mechanisms. In this section, a number of weight loss interventions including traditional preventions of diet, exercise, and pharmacology [40, 121, 125], bariatric surgery [126], and non-invasive neuromodulations of tDCS/TMS and neurofeedback [132–134] will be discussed in detail (Suppl Table 2).

### MRI studies related to bariatric surgery

Bariatric surgery is well known to decrease appetite and hunger and promote satiety, and it is also suspected to increase energy expenditure. Roux-en-Y gastric bypass (RYGB) and laparoscopic sleeve gastrectomy (LSG) are the most widely used procedures in clinical practice, with similar long-term weight loss efficacy [126]. In RYGB, a small gastric pouch is connected to the small intestine, bypassing the stomach, duodenum, and the proximal part of the jejunum, which is a restrictive and reversible procedure [126]. In LSG, the fundus of the stomach is vertically resected and a tube-shaped remnant is left along the lesser curvature [126]. Therapeutic benefits are partly mediated through its actions on the central nervous system. Since weight-loss by other means leads to increased appetite and calorie conservation, the reduced appetite seen after bariatric surgery has been attributed to changes in gut hormones and neuropeptides that influence satiety signals in the brain. It also has a great impact on food preference and cognitive function by altering functional and structural frontal-mesolimbic circuitry.

#### Neural responsive studies

##### Food cues

Food cue reactivity tasks have also been adopted to test surgery-induced brain responses. After LSG in response to high vs. low calorie or high vs. low dense food cues, obese participants showed decreased activation in reward-related areas (caudate, putamen, NAc, pallidum, AMY), and reduction in ghrelin levels was associated with less craving for high-calorie food cues and reduction in DLPFC activation along with strengthened connectivity with vACC a region important for self-control and executive functions at one month post-surgery [129, 179], suggesting a critical role of ghrelin in brain reactivity and eating behaviors. However, two studies reported increased activation in DMPFC and DLPFC, with DLPFC’s activation increasing more in RYGB than LSG at 4- and 12-months post-BS in response to high- vs. low-calorie food cues or during the desire for palatable food regulation vs. desire for palatable food enhancement [180, 181]. Discrepant results across studies might be due to differences in the surgeries used (RYGB vs. LSG), and/or different experimental designs and time points post-surgery. Zoon et al. used a Go/No-Go task with high vs. low energy foods cues to examine surgery-induced cognitive changes; RYGB increased activation in inhibitory control regions (i.e., DLPFC, MPFC, MCC and IFG) in response inhibition to high energy food cues; and decreased activation in regions implicated in metabolic regulation (i.e., STG, PHIPP, and hypothalamus) in response inhibition to low energy food cues [182]. These findings suggest greater cognitive dietary inhibition and decreased rewarding effects related to high-calorie or high-dense food cues.

#### Resting-state fMRI studies

Since obesity is associated with altered RSFC, researchers have assessed whether weight loss after BS can reverse these changes and if that predicts the efficacy of the interventions. After surgery, obese participants showed increased resting-state activity in the DLPFC, SFG, ITG, visual cortex, PCC; and decreased activity in the claustrum, precentral gyrus, putamen, insula, thalamus, and HIPP [127, 183–185]. BS also increased RSFC of VMPFC-DLPFC, PCC/precuneus-caudate/DLPFC, HIPP-insula which is affected by ghrelin, reward network-MPFC, mediodorsal thalamic nucleus (MD)-precuneus/habenular; and decreased RSFC of VMPFC-HIPP/PHIPP, putamen-lateral hypothalamus, between regions involved in food-related saliency attribution and reward-driven eating behavior, and the nodes within and between DMN, SN, and FPN [127, 186–192]. These latter RSFC patterns appear to be normalizing a pre-BS hyperconnected state, which might alter control of eating behavior. Interestingly, baseline activity within the mesolimbic pathway [180], RSFC of NAc-insula, hypothalamus-precentral gyrus, habenular-MD, regions linked to emotional control and social interaction, as well as brain networks related to salience, reward, self-referential, and cognitive processing are associated with reduced BMI [186, 187, 192, 193], providing evidence that specific resting-state activity and/or RSFC patterns might be useful as neuroimaging biomarkers to predict individual weight loss.

#### Structural MRI studies

Obese individuals show marked changes in both GM and WM integrity in various brain regions, and various studies investigated whether BS reversed brain structural alterations. Surprisingly, improvements in cortical morphometry, GM volume, and WM integrity were observed just one-month after LSG [131, 194, 195], and these structural changes sustain through four, six, and even 12 months after LSG or RYGB along with weight loss [130, 156, 194, 196–199]. BS decreased precuneus cortical thickness in association with reduced BMI and increased cortical thickness in the MFG, SFG, STG, vACC, as well as cortical volume in the vACC and postcentral gyrus, which are implicated in executive-control and self-referential processing [195]. After BS, obese participants showed significant increases in FA in the anterior corona radiate, body CC, genu CC, fornix and sagittal stratum (SS), and GM density/volume in the caudate, INS, HIPP/PHIPP, AMY, IFG, SFG, rACC, DMPFC, ITG, MTG, PCC, FFA, and postcentral gyrus, and WM density in the cerebellum, brain stem, CC when compared with those before surgery [130, 131, 194, 198, 199]. In addition to these findings from whole brain analysis, two recent papers focused on changes in structural connectivity between the habenula and the insula induced by LSG. LSG increased connectivity between habenula and homeostatic/hedonic regions including hypothalamus, SFG, AMY and OFC, and habenular-hypothalamus/AMY structural connectivity in association with weight loss at multiple timepoints (1- and 12-month), which also correlated with external and emotional eating [156]. The findings highlight the importance of circuits mediating reward, interoception, and negative emotional processing in the long-term therapeutic benefits of BS. Another study examined the long-term impact of LSG on insula-related structural connectivity, and results showed increased FA/AD of insula-ACC/putamen/caudate, ACC-PCC/precuneus at 12-month post LSG, and FA/AD of insula-ACC were associated with BMI and external eating, respectively [196].

#### Multi-modal MRI studies

Few multi-modal MRI studies have investigated surgery-induced brain functional and structural alterations simultaneously. One study used RS-fMRI, sMRI and DTI at 6- and 12-month after RYGB, and results showed the regions with increased GM/WM density including cerebral cortex of all lobes were associated with elevated regional homogeneity [200]. Hu et al. adopted cue reactivity fMRI task with DTI, and results showed sustained increases in functional and structural connectivity of DLPFC-ACC at 1- and 6-month after LSG, at which time connectivity strength in these regions did not differ from that in normal weight participants [128, 201]. Reduction in BMI correlated with increased FC of right DLPFC-ACC at one month and with increased SC of DLPFC-ACC at one and six months post-LSG. Reduction in craving for high-calorie food cues correlated negatively with increased FC of DLPFC-ACC at six months post-LSG, suggesting that greater prefrontal FC contributes to successful weight loss and reductions in food-cue craving, which each have a distinct temporal course post-LSG. In addition, SC of DLPFC-ACC mediated the relationship between lower ghrelin levels and greater cognitive-control. These findings provide evidence that LSG improved functional and structural connectivity in prefrontal regions, which contribute to enhanced cognitive control and sustained weight loss following surgery.

These brain functional and structural recoveries as revealed by MRI suggest that BS-induced changes in the gut, gut hormones, and circulating peptides along with inflammation may play critical roles in regulating brain functions and structures. Changes in gut hormones and peptide levels after BS are related to weight loss and brain functional and structural changes, and play a role in the regulation of energy homeostasis. In particular, the decrease of ghrelin after LSG due to the removal of the gastric fundus (where ghrelin is mainly produced) directly influences the brain. Ghrelin was associated with less cravings to high-calorie food and reduction in DLPFC activation to food cues along with strengthened connectivity between regions important for self-control and executive functions. Ghrelin also directly affects the HIPP by modulating its connectivity with the INS, implying that ghrelin influences brain reactivity and eating behaviors. Recovery of obesity-related brain GM volume might be due to a reduction of inflammatory cytokines and less metabolic stress, and recovery of WM might be due to remyelination [202]. These data collectively can inform medical professionals performing BS, as studies are now showing the procedure’s power to reverse pathophysiological changes in brain circuits to reverse obesity and associated metabolic diseases. Understanding of these pathways through bariatric surgery research could also pave the way for the design of newer treatments (i.e., knifeless surgery) to combat the obesity epidemic.

### Dietary and Lifestyle Interventions

Dietary and lifestyle interventions aimed at decreasing energy intake and increasing energy expenditure are also an essential component of all weight management programs. Dietary interventions showed obese participants exhibited decreased activations in regions involved with reward processing (i.e., NAc, caudate, putamen, pallidum, MOFC) which related to decreased ghrelin levels and BMI, and increased activation in frontal and temporal cortices when exposed to high vs. low dense energy/protein [40, 203, 204]; dietary interventions also decreased GM volume in the precentral gyrus and insula [65]. A 12-month diet and exercise program increased cerebral blood flow in widespread regions including frontoparietal cortices and subcortical regions [205]. Exercise increased functional connectivity between anterior HIPP and frontal cortex, between PCC and networks including ECN, BG, DMN, between sensory and motor cortical regions, and between networks associated with behavior emergence, self-regulation, and bodily awareness [121, 206–208]. Exercise also decreased mean WM hyperintensity volume and increased global FA value, indicating greater structural integrity of fiber tracts across the brain [209, 210]. In sum, dietary and lifestyle intervention may reduce the adverse impact of obesity on brain function and structure, and these intriguing results have implications for understanding the mechanism behind dietary and lifestyle intervention efficacy.

### Pharmacological treatment

In the search for new obesity treatments, researchers examined the potential of therapies that target adipose tissue-derived and gut-derived hormones; however, these attempts often did not succeed, with the except of glucagon-like peptide-1 (GLP-1) [211]. GLP-1 is secreted after food ingestion from enteroendocrine L cells located in the distal jejunum and ileum [212], and is known mainly for its glucose-lowering effects, as it augments meal-related insulin secretion from the pancreas [213]. Over the past several decades, GLP-1-based therapies have been developed successfully for the treatment of diabetes. Interestingly, besides affecting glucose regulation, the administration of GLP-1 receptor agonists (GLP-1RAs), such as liraglutide, is consistently associated with weight loss [214]. Based on this, several studies performed short- and/or long-term administration of GLP-IRA and examined brain responses to food cues before and after treatment in obese participants [125, 211, 215, 216]. Liraglutide treatment decreased brain activation in reward processing region (i.e., caudate, putamen, AMY, insula, OFC) in obese individuals (with and without type 2 diabetes) in response to viewing food pictures.

Over the last years, the human brain has been variously identified as an insulin-sensitive organ [217]. While insulin influences activity in specific brain areas in some individuals, others experience attenuated or even absent responses, suggestive of brain insulin resistance [217, 218]. This phenomenon was first observed in overweight persons, who not only appeared to be resistant to brain insulin actions in terms of regional brain activity but also with regard to many functional consequences: While insulin in the brain influences food intake and body weight [217, 219] in lean persons, no such effects have been observed in overweight and obese individuals [217, 220]. However, insulin resistance of the brain does not appear to negatively affect all brain functions in overweight subjects since the hormone improves memory consolidation regardless of body weight [220]. This might reflect the fact that insulin resistance differentially affects specific brain areas [221]. Recent studies also used intranasal insulin administration to induce brain insulin action and results showed that insulin increased functional connectivity between prefrontal regions of DMN and HIPP and hypothalamus [222, 223], and the larger the dose of intranasal insulin, the stronger the effects on brain activity (a significant linear decrease with insulin dose was observed in the caudate and hypothalamus) [123]. Further, intranasal neuropeptide oxytocin (OXT), which is a nine-amino acid neuropeptide hormone predominantly synthesized in the paraventricular and supraoptic nuclei of the hypothalamus that decreases food intake and body weight in animal models, had the following effects: (1) reduced food craving during a cognitive control task; (2) reduced functional connectivity between VTA and insula/SMA/AMY/HIPP/MTG during viewing high-calorie food cues; and (3) increased activation in MFG, SFG, precuneus and cingulate cortex to high-calorie food cues [124, 224]. Dapagliflozin, a sodium-glucose cotransporter 2 inhibitor, decreased food cue-related activation in the caudate, insula, AMY after 10 days treatment and in the insula after 16 weeks, whereas exenatide, a GLP-1RA, increased activation in the putamen only after 10 days, and dapagliflozin plus exenatide relative to dapagliflozin only increased activation in the insula and AMY to low-calorie food cues [225]. Finally, bupropion when combined with naltrexone decreased resting-state activity in the MFG and SPG, and increased functional connectivity of SPG-dACC/insula, and decreased functional connectivity of SPG-IFG [122]. In general, these pharmacology studies aim to target specific brain regions or circuitries that play a critical role in food-intake control and regulation.

### Neuromodulation/neurofeedback

Based on extensive findings on the neurocircuitry underlying obesity, promising neuromodulation techniques including non-invasive repetitive transcranial magnetic stimulation (rTMS), transcranial direct current stimulation (tDCS), and real-time neurofeedback have been used to regulate brain activity and improve healthy eating behaviors [132–134, 226, 227]. In a recent imaging study, rTMS induced weight loss, increased betweenness centrality (the degree to which a region influences information flow across the brain, based on how many other regions have short paths connecting to it) within FPN and MOFC, and decreased degree centrality in the occipital pole [134, 226]. In addition, DMPFC rTMS increased activation over the stimulation site during a delay discounting task [227]. tDCS over DLPFC decreased activation in the ventral striatum in responses to food cue [133]. Neurofeedback training increased functional connectivity between DLPFC and VMPFC, a circuit involved with processing food value during self-regulation compared to passive viewing [132, 228]. At this moment, these interventions remain largely experimental, but growing understanding of the mechanisms involved, together with the rising number of studies in this area, means that the clinical utility of these interventions is likely to become clearer soon.

## Perspectives

Recent MRI studies help identify obesity-related brain functional and structural abnormalities and unravel the underlying neural mechanisms of obesity and of therapeutic interventions. There are several common limitations of these studies. Firstly, the sample sizes are relatively small, and they often do not exclude participants with metabolic diseases, which could contribute to changes in brain and behavior beyond obesity alone. Secondly, a variety of experimental designs including food cues, taste, and smell were employed, and this may contribute to differences in results across studies. Thirdly, studies varied in their data analysis approach and statistical thresholds. In addition, the scope of the current review is limited to mainly MRI-related reports within the past five years, however, obesity is associated with a host of neurobiological changes that could be captured with other technologies such as positron emission tomography imaging.

Despite bariatric surgery being the most effective treatment for obesity, a proportion of participants have suboptimal weight loss post-surgery, including an estimated one-third of individuals who do not maintain long-term weight loss [229]. Further, among those who do lose weight, there is tremendous variability in efficacy, ranging from 5% to 55% total weight loss at three years post-surgery [230]. Thus, it is important to understand the mechanisms behind the variance in weight loss and identify baseline biomarkers to predict optimal weight loss with BS to screen obese patients suitable for surgery. In addition, post-surgical weight rebound is a challenge for both obese patients and the surgeon. A 5-year follow-up study indicated that the median percentage of maximum weight loss was 37.4% of presurgery weight and occurred a median of 2.0 years after RYGB; rate of weight regain was highest during the first year after reaching nadir weight, but weight gain continued throughout follow-up: 5 years after nadir weight, 43.6% regained 5 BMI points; 50.2% regained 15% of nadir weight; and 67.3% regained 20% of maximum weight lost [231]. To address this major challenge, longer-term neuroimaging studies on brain function and structure as well as measurement of other potential biomarkers (i.e., gut hormones, adipokines, microbiota) are needed to better understand these outcomes. Combining other interventions/treatments including neuromodulation/neurofeedback could also be a promising strategy to combat suboptimal outcomes.

## Conclusion

Overall, obesity is associated with brain functional and structural alterations, including neural responses to external stimuli (i.e., food cues, taste and smell), resting-state activity and functional connectivity, brain activation, and functional connectivity during cognitive tasks (i.e., impulsive decision-making, inhibitory control, learning/memory, and attention), and GM/WM volume and WM integrity. Weight loss interventions, particularly BS can normalize these various pathophysiological processes. Neuroimaging research is poised to improve understanding of the pathophysiology of obesity and thereby guide effective treatment and prevention efforts.

## Supplementary information

Supplementary Table 1

Supplementary Table 2

## References

[1] Jaacks LM, Vandevijvere S, Pan A, McGowan CJ, Wallace C, Imamura F (2019). The obesity transition: stages of the global epidemic. Lancet Diabetes Endocrinol.

[2] WHO. Fact sheet: obesity and overweight. February 2018 2018-10-20: http://www.who.int/news-room/fact-sheets/detail/obesity-and-overweight.

[3] Obesity in China: time to act. Lancet Diabetes Endocrinol. 2021;7**:**231–40 https://www.thelancet.com/journals/landia/article/PIIS2213-8587(21)00150-9/fulltext. 10.1016/S2213-8587(21)00150-9 34097870

[4] Wang L, Zhou B, Zhao Z, Yang L, Zhang M, Jiang Y (2021). Body-mass index and obesity in urban and rural China: findings from consecutive nationally representative surveys during 2004-18. Lancet.

[5] Wang Y, Zhao L, Gao L, Pan A, Xue H (2021). Health policy and public health implications of obesity in China. Lancet Diabetes Endocrinol.

[6] Collaboration NRF (2016). Trends in adult body-mass index in 200 countries from 1975 to 2014: a pooled analysis of 1698 population-based measurement studies with 19.2 million participants. Lancet.

[7] Ralston J, Brinsden H, Buse K, Candeias V, Caterson I, Hassell T (2018). Time for a new obesity narrative. Lancet.

[8] Ma Y, Ajnakina O, Steptoe A, Cadar D (2020). Higher risk of dementia in English older individuals who are overweight or obese. Int J Epidemiol.

[9] De Silva A, Salem V, Matthews PM, Dhillo WS (2012). The use of functional MRI to study appetite control in the CNS. Exp Diabetes Res.

[10] Anstey KJ, Cherbuin N, Budge M, Young J (2011). Body mass index in midlife and late-life as a risk factor for dementia: a meta-analysis of prospective studies. Obes Rev.

[11] Fox MD, Raichle ME (2007). Spontaneous fluctuations in brain activity observed with functional magnetic resonance imaging. Nat Rev Neurosci.

[12] Belfort-DeAguiar R, Seo D, Lacadie C, Naik S, Schmidt C, Lam W (2018). Humans with obesity have disordered brain responses to food images during physiological hyperglycemia. Am J Physiol Endocrinol Metab.

[13] Blechert J, Klackl J, Miedl SF, Wilhelm FH (2016). To eat or not to eat: Effects of food availability on reward system activity during food picture viewing. Appetite.

[14] Bogdanov VB, Bogdanova OV, Dexpert S, Delgado I, Beyer H, Aubert A (2020). Reward-related brain activity and behavior are associated with peripheral ghrelin levels in obesity. Psychoneuroendocrinology.

[15] Bohon C (2017). Brain response to taste in overweight children: A pilot feasibility study. PLoS One.

[16] Carnell S, Benson L, Chang KV, Wang Z, Huo Y, Geliebter A (2017). Neural correlates of familial obesity risk and overweight in adolescence. Neuroimage.

[17] Chin SH, Kahathuduwa CN, Stearns MB, Davis T, Binks M (2018). Is hunger important to model in fMRI visual food-cue reactivity paradigms in adults with obesity and how should this be done?. Appetite.

[18] Demos KE, Sweet LH, Hart CN, McCaffery JM, Williams SE, Mailloux KA (2017). The effects of experimental manipulation of sleep duration on neural response to food cues. Sleep.

[19] Dodd SL, Long JD, Hou J, Kahathuduwa CN, O’Boyle MW (2020). Brain activation and affective judgements in response to personal dietary images: An fMRI preliminary study. Appetite.

[20] Ebrahimi C, Koch SP, Pietrock C, Fydrich T, Heinz A, Schlagenhauf F (2019). Opposing roles for amygdala and vmPFC in the return of appetitive conditioned responses in humans. Transl Psychiatry.

[21] Gearhardt AN, Yokum S, Harris JL, Epstein LH, Lumeng JC (2020). Neural response to fast food commercials in adolescents predicts intake. Am J Clin Nutr.

[22] Geha P, Cecchi G, Todd CR, Abdallah C, Small DM (2017). Reorganization of brain connectivity in obesity. Hum Brain Mapp.

[23] Han P, Roitzsch C, Horstmann A, Possel M, Hummel T (2021). Increased brain reward responsivity to food-related odors in obesity. Obesity.

[24] Jacobson A, Green E, Haase L, Szajer J, Murphy C (2019). Differential effects of BMI on brain response to odor in olfactory, reward and memory regions: evidence from fMRI. Nutrients.

[25] Masterson TD, Bermudez MA, Austen M, Lundquist E, Pearce AL, Bruce AS (2019). Food commercials do not affect energy intake in a laboratory meal but do alter brain responses to visual food cues in children. Appetite.

[26] Puzziferri N, Zigman JM, Thomas BP, Mihalakos P, Gallagher R, Lutter M (2016). Brain imaging demonstrates a reduced neural impact of eating in obesity. Obesity.

[27] Shearrer GE, Stice E, Burger KS (2018). Adolescents at high risk of obesity show greater striatal response to increased sugar content in milkshakes. Am J Clin Nutr.

[28] Stopyra MA, Friederich HC, Lavandier N, Monning E, Bendszus M, Herzog W (2021). Homeostasis and food craving in obesity: a functional MRI study. Int J Obes (Lond).

[29] Veit R, Horstman LI, Hege MA, Heni M, Rogers PJ, Brunstrom JM (2020). Health, pleasure, and fullness: changing mindset affects brain responses and portion size selection in adults with overweight and obesity. Int J Obes.

[30] Wiemerslage L, Nilsson EK, Solstrand DL, Ence-Eriksson F, Castillo S, Larsen AL (2016). An obesity-associated risk allele within the FTO gene affects human brain activity for areas important for emotion, impulse control and reward in response to food images. Eur J Neurosci.

[31] Winter SR, Yokum S, Stice E, Osipowicz K, Lowe MR (2017). Elevated reward response to receipt of palatable food predicts future weight variability in healthy-weight adolescents. Am J Clin Nutr.

[32] Morys F, Bode S, Horstmann A (2018). Dorsolateral and medial prefrontal cortex mediate the influence of incidental priming on economic decision making in obesity. Sci Rep.

[33] Hsu JS, Wang PW, Ko CH, Hsieh TJ, Chen CY, Yen JY (2017). Altered brain correlates of response inhibition and error processing in females with obesity and sweet food addiction: A functional magnetic imaging study. Obes Res Clin Pr.

[34] Kube J, Mathar D, Horstmann A, Kotz SA, Villringer A, Neumann J (2018). Altered monetary loss processing and reinforcement-based learning in individuals with obesity. Brain Imaging Behav.

[35] Janssen LK, Duif I, van Loon I, Wegman J, de Vries J, Cools R (2017). Loss of lateral prefrontal cortex control in food-directed attention and goal-directed food choice in obesity. Neuroimage.

[36] Merchant JS, Cosme D, Giuliani NR, Dirks B, Berkman ET (2020). Neural substrates of food valuation and its relationship with BMI and healthy eating in higher BMI Individuals. Front Behav Neurosci.

[37] Verdejo-Roman J, Fornito A, Soriano-Mas C, Vilar-Lopez R, Verdejo-Garcia A (2017). Independent functional connectivity networks underpin food and monetary reward sensitivity in excess weight. Neuroimage.

[38] Verdejo-Roman J, Vilar-Lopez R, Navas JF, Soriano-Mas C, Verdejo-Garcia A (2017). Brain reward system’s alterations in response to food and monetary stimuli in overweight and obese individuals. Hum Brain Mapp.

[39] Contreras-Rodriguez O, Mata F, Verdejo-Roman J, Ramirez-Bernabe R, Moreno D, Vilar-Lopez R (2020). Neural-based valuation of functional foods among lean and obese individuals. Nutr Res.

[40] Simon JJ, Becker A, Sinno MH, Skunde M, Bendszus M, Preissl H (2018). Neural food reward processing in successful and unsuccessful weight maintenance. Obesity.

[41] Spetter MS, Feld GB, Thienel M, Preissl H, Hege MA, Hallschmid M (2018). Oxytocin curbs calorie intake via food-specific increases in the activity of brain areas that process reward and establish cognitive control. Sci Rep.

[42] Weygandt M, Spranger J, Leupelt V, Maurer L, Bobbert T, Mai K (2019). Interactions between neural decision-making circuits predict long-term dietary treatment success in obesity. Neuroimage.

[43] Zhang W, Li G, Manza P, Hu Y, Wang J, Lv G (2022). Functional abnormality of the executive control network in individuals with obesity during delay discounting. Cereb Cortex.

[44] Cheke LG, Bonnici HM, Clayton NS, Simons JS (2017). Obesity and insulin resistance are associated with reduced activity in core memory regions of the brain. Neuropsychologia.

[45] Zhao J, Long Z, Li Y, Qin Y, Liu Y (2022). Alteration of regional heterogeneity and functional connectivity for obese undergraduates: evidence from resting-state fMRI. Brain Imaging Behav.

[46] Baek K, Morris LS, Kundu P, Voon V (2017). Disrupted resting-state brain network properties in obesity: decreased global and putaminal cortico-striatal network efficiency. Psychol Med.

[47] Beyer F, Kharabian MS, Huntenburg JM, Lampe L, Luck T, Riedel-Heller SG (2017). Higher body mass index is associated with reduced posterior default mode connectivity in older adults. Hum Brain Mapp.

[48] Contreras-Rodriguez O, Martin-Perez C, Vilar-Lopez R, Verdejo-Garcia A (2017). Ventral and dorsal striatum networks in obesity: link to food craving and weight gain. Biol Psychiatry.

[49] Ding Y, Ji G, Li G, Zhang W, Hu Y, Liu L (2020). Altered interactions among resting-state networks in individuals with obesity. Obesity.

[50] Gupta A, Mayer EA, Labus JS, Bhatt RR, Ju T, Love A (2018). Sex commonalities and differences in obesity-related alterations in intrinsic brain activity and connectivity. Obesity.

[51] Legget KT, Wylie KP, Cornier MA, Berman BD, Tregellas JR (2021). Altered between-network connectivity in individuals prone to obesity. Physiol Behav.

[52] Meng Q, Han Y, Ji G, Li G, Hu Y, Liu L (2018). Disrupted topological organization of the frontal-mesolimbic network in obese patients. Brain Imaging Behav.

[53] Moreno-Lopez L, Contreras-Rodriguez O, Soriano-Mas C, Stamatakis EA, Verdejo-Garcia A (2016). Disrupted functional connectivity in adolescent obesity. Neuroimage Clin.

[54] Nakamura Y, Ikuta T (2017). Caudate-precuneus functional connectivity is associated with obesity preventive eating tendency. Brain Connect.

[55] Park BY, Byeon K, Lee MJ, Chung CS, Kim SH, Morys F (2020). Whole-brain functional connectivity correlates of obesity phenotypes. Hum Brain Mapp.

[56] Park BY, Seo J, Park H (2016). Functional brain networks associated with eating behaviors in obesity. Sci Rep.

[57] Rashid B, Dev SI, Esterman M, Schwarz NF, Ferland T, Fortenbaugh FC (2019). Aberrant patterns of default-mode network functional connectivity associated with metabolic syndrome: A resting-state study. Brain Behav.

[58] Ravichandran S, Bhatt RR, Pandit B, Osadchiy V, Alaverdyan A, Vora P (2021). Alterations in reward network functional connectivity are associated with increased food addiction in obese individuals. Sci Rep.

[59] Shapiro A, Johnson SL, Sutton B, Legget KT, Dabelea D, Tregellas JR (2019). Eating in the absence of hunger in young children is related to brain reward network hyperactivity and reduced functional connectivity in executive control networks. Pediatr Obes.

[60] Tan Z, Li G, Zhang W, Wang J, Hu Y, Li H (2021). Obese individuals show disrupted dynamic functional connectivity between basal ganglia and salience networks. Cereb Cortex.

[61] Zhang P, Liu Y, Lv H, Li MY, Yu FX, Wang Z (2019). Integration of neural reward processing and appetite-related signaling in obese females: evidence from resting-state fMRI. J Magn Reson Imaging.

[62] Zhang P, Liu Y, Yu FX, Wu GW, Li MY, Wang Z (2021). Hierarchical integrated processing of reward-related regions in obese males: A graph-theoretical-based study. Appetite.

[63] Zhang P, Wu GW, Yu FX, Liu Y, Li MY, Wang Z (2020). Abnormal regional neural activity and reorganized neural network in obesity: evidence from resting-state fMRI. Obesity.

[64] Pflanz CP, Tozer DJ, Harshfield EL, Tay J, Farooqi S, Markus HS (2022). Central obesity is selectively associated with cerebral gray matter atrophy in 15,634 subjects in the UK Biobank. Int J Obes.

[65] Honea RA, Szabo-Reed AN, Lepping RJ, Perea R, Breslin F, Martin LE (2016). Voxel-based morphometry reveals brain gray matter volume changes in successful dieters. Obesity.

[66] Kakoschke N, Lorenzetti V, Caeyenberghs K, Verdejo-Garcia A (2019). Impulsivity and body fat accumulation are linked to cortical and subcortical brain volumes among adolescents and adults. Sci Rep.

[67] Ludwig M, Richter M, Goltermann J, Redlich R, Repple J, Flint C (2021). Novelty seeking is associated with increased body weight and orbitofrontal grey matter volume reduction. Psychoneuroendocrinology.

[68] Migueles JH, Cadenas-Sanchez C, Esteban-Cornejo I, Mora-Gonzalez J, Rodriguez-Ayllon M, Solis-Urra P (2021). Associations of sleep with gray matter volume and their implications for academic achievement, executive function and intelligence in children with overweight/obesity. Pediatr Obes.

[69] Migueles JH, Martinez-Nicolas A, Cadenas-Sanchez C, Esteban-Cornejo I, Muntaner-Mas A, Mora-Gonzalez J (2021). Activity-rest circadian pattern and academic achievement, executive function, and intelligence in children with obesity. Scand J Med Sci Sports.

[70] Mokhtari F, Paolini BM, Burdette JH, Marsh AP, Rejeski WJ, Laurienti PJ (2016). Baseline gray- and white-matter volume predict successful weight loss in the elderly. Obesity.

[71] Nouwen A, Chambers A, Chechlacz M, Higgs S, Blissett J, Barrett TG (2017). Microstructural abnormalities in white and gray matter in obese adolescents with and without type 2 diabetes. Neuroimage Clin.

[72] Opel N, Redlich R, Kaehler C, Grotegerd D, Dohm K, Heindel W (2017). Prefrontal gray matter volume mediates genetic risks for obesity. Mol Psychiatry.

[73] Parcet MA, Adrian-Ventura J, Costumero V, Avila C (2020). Individual differences in hippocampal volume as a function of BMI and reward sensitivity. Front Behav Neurosci.

[74] Perlaki G, Molnar D, Smeets P, Ahrens W, Wolters M, Eiben G (2018). Volumetric gray matter measures of amygdala and accumbens in childhood overweight/obesity. PLoS One.

[75] Sweat V, Yates KF, Migliaccio R, Convit A (2017). Obese adolescents show reduced cognitive processing speed compared with healthy weight peers. Child Obes.

[76] Thapaliya G, Chen L, Jansen E, Smith KR, Sadler JR, Benson L (2021). Familial obesity risk and current excess weight influence brain structure in adolescents. Obesity.

[77] Tungler A, Van der Auwera S, Wittfeld K, Frenzel S, Terock J, Roder N (2021). Body mass index but not genetic risk is longitudinally associated with altered structural brain parameters. Sci Rep.

[78] Turan S, Sarioglu FC, Erbas IM, Cavusoglu B, Karagoz E, Sisman AR (2021). Altered regional grey matter volume and appetite-related hormone levels in adolescent obesity with or without binge-eating disorder. Eat Weight Disord.

[79] Wang H, Wen B, Cheng J, Li H (2017). Brain structural differences between normal and obese adults and their links with lack of perseverance, negative urgency, and sensation seeking. Sci Rep.

[80] Yokum S, Stice E (2017). Initial body fat gain is related to brain volume changes in adolescents: A repeated-measures voxel-based morphometry study. Obesity.

[81] Opel N, Thalamuthu A, Milaneschi Y, Grotegerd D, Flint C, Leenings R (2021). Brain structural abnormalities in obesity: relation to age, genetic risk, and common psychiatric disorders: Evidence through univariate and multivariate mega-analysis including 6420 participants from the ENIGMA MDD working group. Mol Psychiatry.

[82] Beyer F, Garcia-Garcia I, Heinrich M, Schroeter ML, Sacher J, Luck T (2019). Neuroanatomical correlates of food addiction symptoms and body mass index in the general population. Hum Brain Mapp.

[83] Medic N, Ziauddeen H, Ersche KD, Farooqi IS, Bullmore ET, Nathan PJ (2016). Increased body mass index is associated with specific regional alterations in brain structure. Int J Obes (Lond).

[84] Ronan L, Alexander-Bloch A, Fletcher PC (2020). Childhood obesity, cortical structure, and executive function in healthy children. Cereb Cortex.

[85] Saute RL, Soder RB, Alves FJ, Baldisserotto M, Franco AR (2018). Increased brain cortical thickness associated with visceral fat in adolescents. Pediatr Obes.

[86] Solis-Urra P, Esteban-Cornejo I, Rodriguez-Ayllon M, Verdejo-Roman J, Labayen I, Catena A (2022). Early life factors and white matter microstructure in children with overweight and obesity: The ActiveBrains project. Clin Nutr.

[87] Estella NM, Sanches LG, Maranhao MF, Hoexter MQ, Schmidt U, Campbell IC (2020). Brain white matter microstructure in obese women with binge eating disorder. Eur Eat Disord Rev.

[88] Kullmann S, Callaghan MF, Heni M, Weiskopf N, Scheffler K, Haring HU (2016). Specific white matter tissue microstructure changes associated with obesity. Neuroimage.

[89] Ottino-Gonzalez J, Jurado MA, Garcia-Garcia I, Segura B, Marques-Iturria I, Sender-Palacios MJ (2018). Allostatic load and disordered white matter microstructure in overweight adults. Sci Rep.

[90] Papageorgiou I, Astrakas LG, Xydis V, Alexiou GA, Bargiotas P, Tzarouchi L (2017). Abnormalities of brain neural circuits related to obesity: A Diffusion Tensor Imaging study. Magn Reson Imaging.

[91] Repple J, Opel N, Meinert S, Redlich R, Hahn T, Winter NR (2018). Elevated body-mass index is associated with reduced white matter integrity in two large independent cohorts. Psychoneuroendocrino.

[92] Reyes S, Rimkus CM, Lozoff B, Biswal BB, Peirano P, Algarin C (2020). Assessing cognitive control and the reward system in overweight young adults using sensitivity to incentives and white matter integrity. PLoS One.

[93] Spindler M, Ozyurt J, Thiel CM (2020). Automated diffusion-based parcellation of the hypothalamus reveals subunit-specific associations with obesity. Sci Rep.

[94] Takeuchi H, Taki Y, Nouchi R, Yokoyama R, Nakagawa S, Iizuka K (2020). The associations of BMI with mean diffusivity of basal ganglia among young adults with mild obesity and without obesity. Sci Rep.

[95] van Bloemendaal L, Ijzerman RG, Ten KJ, Barkhof F, Diamant M, Veltman DJ (2016). Alterations in white matter volume and integrity in obesity and type 2 diabetes. Metab Brain Dis.

[96] Zhang R, Beyer F, Lampe L, Luck T, Riedel-Heller SG, Loeffler M (2018). White matter microstructural variability mediates the relation between obesity and cognition in healthy adults. Neuroimage.

[97] Medic N, Kochunov P, Ziauddeen H, Ersche KD, Nathan PJ, Ronan L (2019). BMI-related cortical morphometry changes are associated with altered white matter structure. Int J Obes.

[98] Adise S, Allgaier N, Laurent J, Hahn S, Chaarani B, Owens M (2021). Multimodal brain predictors of current weight and weight gain in children enrolled in the ABCD study (R). Dev Cogn Neurosci.

[99] Alarcon G, Ray S, Nagel BJ (2016). Lower working memory performance in overweight and obese adolescents is mediated by white matter microstructure. J Int Neuropsychol Soc.

[100] Augustijn M, Di Biase MA, Zalesky A, Van Acker L, De Guchtenaere A, D’Hondt E (2019). Structural connectivity and weight loss in children with obesity: a study of the “connectobese”. Int J Obes.

[101] Burdette JH, Laurienti PJ, Miron LL, Bahrami M, Simpson SL, Nicklas BJ (2020). Functional brain networks: unique patterns with hedonic appetite and confidence to resist eating in older adults with obesity. Obesity.

[102] Byeon K, Park BY, Park H (2019). Spatially guided functional correlation tensor: A new method to associate body mass index and white matter neuroimaging. Comput Biol Med.

[103] Chao SH, Liao YT, Chen VC, Li CJ, McIntyre RS, Lee Y (2018). Correlation between brain circuit segregation and obesity. Behav Brain Res.

[104] Chen PA, Chavez RS, Heatherton TF (2017). Structural integrity between executive control and reward regions of the brain predicts body fat percentage in chronic dieters. Cogn Neurosci.

[105] de Groot CJ, van den Akker E, Rings E, Delemarre-van DWH, van der Grond J (2017). Brain structure, executive function and appetitive traits in adolescent obesity. Pediatr Obes.

[106] Donofry SD, Jakicic JM, Rogers RJ, Watt JC, Roecklein KA, Erickson KI (2020). Comparison of food cue-evoked and resting-state functional connectivity in obesity. Psychosom Med.

[107] Figley CR, Asem JS, Levenbaum EL, Courtney SM (2016). Effects of body mass index and body fat percent on default mode, executive control, and salience network structure and function. Front Neurosci.

[108] Gogniat MA, Robinson TL, Mewborn CM, Jean KR, Miller LS (2018). Body mass index and its relation to neuropsychological functioning and brain volume in healthy older adults. Behav Brain Res.

[109] Gupta A, Mayer EA, Hamadani K, Bhatt R, Fling C, Alaverdyan M (2017). Sex differences in the influence of body mass index on anatomical architecture of brain networks. Int J Obes.

[110] Ho MC, Chen VC, Chao SH, Fang CT, Liu YC, Weng JC (2018). Neural correlates of executive functions in patients with obesity. Peerj.

[111] Li G, Hu Y, Zhang W, Ding Y, Wang Y, Wang J (2021). Resting activity of the hippocampus and amygdala in obese individuals predicts their response to food cues. Addict Biol.

[112] Luo X, Li K, Jia YL, Zeng Q, Jiaerken Y, Qiu T (2018). Abnormal of inter-hemispheric functional connectivity in elderly subjects with overweight/obesity. Obes Res Clin Pr.

[113] Mestre ZL, Bischoff-Grethe A, Eichen DM, Wierenga CE, Strong D, Boutelle KN (2017). Hippocampal atrophy and altered brain responses to pleasant tastes among obese compared with healthy weight children. Int J Obes.

[114] Park BY, Lee MJ, Kim M, Kim SH, Park H (2018). Structural and functional brain connectivity changes between people with abdominal and non-abdominal obesity and their association with behaviors of eating disorders. Front Neurosci.

[115] Singh MK, Leslie SM, Packer MM, Zaiko YV, Phillips OR, Weisman EF (2019). Brain and behavioral correlates of insulin resistance in youth with depression and obesity. Horm Behav.

[116] Syan SK, Owens MM, Goodman B, Epstein LH, Meyre D, Sweet LH (2019). Deficits in executive function and suppression of default mode network in obesity. Neuroimage Clin.

[117] Thomas K, Beyer F, Lewe G, Zhang R, Schindler S, Schonknecht P (2019). Higher body mass index is linked to altered hypothalamic microstructure. Sci Rep.

[118] Timper K, Bruning JC (2017). Hypothalamic circuits regulating appetite and energy homeostasis: pathways to obesity. Dis Model Mech.

[119] Koliaki C, Liatis S, Dalamaga M, Kokkinos A (2020). The implication of gut hormones in the regulation of energy homeostasis and their role in the pathophysiology of obesity. Curr Obes Rep.

[120] Rossi MA, Stuber GD (2018). Overlapping brain circuits for homeostatic and hedonic feeding. Cell Metab.

[121] Esteban-Cornejo I, Stillman CM, Rodriguez-Ayllon M, Kramer AF, Hillman CH, Catena A (2021). Physical fitness, hippocampal functional connectivity and academic performance in children with overweight/obesity: The ActiveBrains project. Brain Behav Immun.

[122] Wang GJ, Zhao J, Tomasi D, Kojori ES, Wang R, Wiers CE (2018). Effect of combined naltrexone and bupropion therapy on the brain’s functional connectivity. Int J Obes (Lond).

[123] Kullmann S, Veit R, Peter A, Pohmann R, Scheffler K, Haring HU (2018). Dose-dependent effects of intranasal insulin on resting-state brain activity. J Clin Endocrinol Metab.

[124] Striepens N, Schroter F, Stoffel-Wagner B, Maier W, Hurlemann R, Scheele D (2016). Oxytocin enhances cognitive control of food craving in women. Hum Brain Mapp.

[125] van Ruiten CC, Ten KJ, van Bloemendaal L, Nieuwdorp M, Veltman DJ, IJzerman RG (2022). Eating behavior modulates the sensitivity to the central effects of GLP-1 receptor agonist treatment: a secondary analysis of a randomized trial. Psychoneuroendocrino.

[126] Peterli R, Wolnerhanssen BK, Peters T, Vetter D, Kroll D, Borbely Y (2018). Effect of laparoscopic sleeve gastrectomy vs laparoscopic Roux-en-Y gastric bypass on weight loss in patients with morbid obesity: The SM-BOSS randomized clinical trial. JAMA.

[127] Zhang Y, Ji G, Li G, Hu Y, Liu L, Jin Q (2019). Ghrelin reductions following bariatric surgery were associated with decreased resting state activity in the hippocampus. Int J Obes.

[128] Hu Y, Ji G, Li G, Manza P, Zhang W, Wang J (2021). Brain connectivity, and hormonal and behavioral correlates of sustained weight loss in obese patients after laparoscopic sleeve gastrectomy. Cereb Cortex.

[129] Li G, Ji G, Hu Y, Liu L, Jin Q, Zhang W (2019). Reduced plasma ghrelin concentrations are associated with decreased brain reactivity to food cues after laparoscopic sleeve gastrectomy. Psychoneuroendocrinology.

[130] Tuulari JJ, Karlsson HK, Antikainen O, Hirvonen J, Pham T, Salminen P (2016). Bariatric surgery induces white and grey matter density recovery in the morbidly obese: a voxel-based morphometric study. Hum Brain Mapp.

[131] Zhang Y, Ji G, Xu M, Cai W, Zhu Q, Qian L (2016). Recovery of brain structural abnormalities in morbidly obese patients after bariatric surgery. Int J Obes (Lond).

[132] Kohl SH, Veit R, Spetter MS, Gunther A, Rina A, Luhrs M (2019). Real-time fMRI neurofeedback training to improve eating behavior by self-regulation of the dorsolateral prefrontal cortex: A randomized controlled trial in overweight and obese subjects. Neuroimage.

[133] Ghobadi-Azbari P, Malmir N, Vartanian M, Mahdavifar-Khayati R, Robatmili S, Hadian V (2022). Transcranial direct current stimulation to modulate brain reactivity to food cues in overweight and obese adults: study protocol for a randomized controlled trial with fMRI (NeuroStim-Obesity). Trials.

[134] Kim SH, Park BY, Byeon K, Park H, Kim Y, Eun YM (2019). The effects of high-frequency repetitive transcranial magnetic stimulation on resting-state functional connectivity in obese adults. Diabetes Obes Metab.

[135] Ghobadi-Azbari P, Mahdavifar KR, Sangchooli A, Ekhtiari H (2022). Task-dependent effective connectivity of the reward network during food cue-reactivity: a dynamic causal modeling investigation. Front Behav Neurosci.

[136] Rapuano KM, Huckins JF, Sargent JD, Heatherton TF, Kelley WM (2016). Individual differences in reward and somatosensory-motor brain regions correlate with adiposity in adolescents. Cereb Cortex.

[137] Ulrich M, Endres F, Kolle M, Adolph O, Widenhorn-Muller K, Gron G (2016). Glucose modulates food-related salience coding of midbrain neurons in humans. Hum Brain Mapp.

[138] Sadler JR, Shearrer GE, Papantoni A, Yokum ST, Stice E, Burger KS. Correlates of neural adaptation to food cues and taste: the role of obesity risk factors. Soc Cogn Affect Neurosci. 2021; 10.1093/scan/nsab018. 10.1093/scan/nsab018 PMC10074771 33681997

[139] Jastreboff AM, Sinha R, Arora J, Giannini C, Kubat J, Malik S (2016). Altered brain response to drinking glucose and fructose in obese adolescents. Diabetes.

[140] Michaud A, Vainik U, Garcia-Garcia I, Dagher A (2017). Overlapping neural endophenotypes in addiction and obesity. Front Endocrinol.

[141] Miranda-Olivos R, Steward T, Martinez-Zalacain I, Mestre-Bach G, Juaneda-Segui A, Jimenez-Murcia S (2021). The neural correlates of delay discounting in obesity and binge eating disorder. J Behav Addict.

[142] Steward T, Menchon JM, Jimenez-Murcia S, Soriano-Mas C, Fernandez-Aranda F (2018). Neural network alterations across eating disorders: a narrative review of fMRI studies. Curr Neuropharmacol.

[143] Volkow ND, Wang GJ, Tomasi D, Baler RD (2013). The addictive dimensionality of obesity. Biol Psychiatry.

[144] Loprinzi PD, Frith E (2018). Obesity and episodic memory function. J Physiol Sci.

[145] Tan Z, Hu Y, Ji G, Li G, Ding Y, Zhang W (2022). Alterations in functional and structural connectivity of basal ganglia network in patients with obesity. Brain Topogr.

[146] Voigt K, Razi A, Harding IH, Andrews ZB, Verdejo-Garcia A (2021). Neural network modelling reveals changes in directional connectivity between cortical and hypothalamic regions with increased BMI. Int J Obes.

[147] Avery JA, Powell JN, Breslin FJ, Lepping RJ, Martin LE, Patrician TM (2017). Obesity is associated with altered mid-insula functional connectivity to limbic regions underlying appetitive responses to foods. J Psychopharmacol.

[148] Hogenkamp PS, Zhou W, Dahlberg LS, Stark J, Larsen AL, Olivo G (2016). Higher resting-state activity in reward-related brain circuits in obese versus normal-weight females independent of food intake. Int J Obes.

[149] Shaw ME, Sachdev PS, Abhayaratna W, Anstey KJ, Cherbuin N (2018). Body mass index is associated with cortical thinning with different patterns in mid- and late-life. Int J Obes.

[150] Franz CE, Xian H, Lew D, Hatton SN, Puckett O, Whitsel N (2019). Body mass trajectories and cortical thickness in middle-aged men: a 42-year longitudinal study starting in young adulthood. Neurobiol Aging.

[151] Bobb JF, Schwartz BS, Davatzikos C, Caffo B (2014). Cross-sectional and longitudinal association of body mass index and brain volume. Hum Brain Mapp.

[152] Arnoldussen IAC, Gustafson DR, Leijsen EMC, de Leeuw FE, Kiliaan AJ (2019). Adiposity is related to cerebrovascular and brain volumetry outcomes in the RUN DMC study. Neurology.

[153] Steward T, Pico-Perez M, Mestre-Bach G, Martinez-Zalacain I, Sunol M, Jimenez-Murcia S (2019). A multimodal MRI study of the neural mechanisms of emotion regulation impairment in women with obesity. Transl Psychiatry.

[154] Dekkers IA, Jansen PR, Lamb HJ (2019). Obesity, brain volume, and white matter microstructure at MRI: A cross-sectional UK Biobank Study. Radiology.

[155] Rapuano KM, Laurent JS, Hagler DJ, Hatton SN, Thompson WK, Jernigan TL (2020). Nucleus accumbens cytoarchitecture predicts weight gain in children. Proc Natl Acad Sci USA.

[156] Wang J, Ji G, Li G, Hu Y, Zhang W, Ji W et al. Habenular connectivity predict weight loss and negative emotional-related eating behavior after laparoscopic sleeve gastrectomy. Cereb Cortex. 2022; 10.1093/cercor/bhac191. 10.1093/cercor/bhac191 PMC10365841 35580853

[157] Doornweerd S, De Geus EJ, Barkhof F, Van Bloemendaal L, Boomsma DI, Van Dongen JV (2018). Brain reward responses to food stimuli among female monozygotic twins discordant for BMI. Brain Imaging Behav.

[158] Carbine KA, Duraccio KM, Kirwan CB, Muncy NM, LeCheminant JD, Larson MJ (2018). A direct comparison between ERP and fMRI measurements of food-related inhibitory control: Implications for BMI status and dietary intake. NeuroImage.

[159] Friedman JM (2019). Leptin and the endocrine control of energy balance. Nat Metab.

[160] Martín MÁ, Ramos S (2021). Dietary Flavonoids and Insulin signaling in diabetes and obesity. Cells.

[161] Steinert RE, Feinle-Bisset C, Asarian L, Horowitz M, Beglinger C, Geary N (2017). Ghrelin, CCK, GLP-1, and PYY(3-36): Secretory Controls and Physiological Roles in Eating and Glycemia in Health, Obesity, and After RYGB. Physiol Rev.

[162] Farooqi IS, O’Rahilly S Genetic Syndromes Associated with Obesity. in: Jameson JL, De Groot LJ (Eds.). Endocrinology: Adult and Pediatric. 7th Edition, Saunders, Elsevier. 2016;491–7.e2.

[163] Cowley MA, Smart JL, Rubinstein M, Cerda’n MG, Diano S, Horvath TL (2001). Leptin activates anorexigenic POMC neurons through a neural network in the arcuate nucleus. Nature.

[164] Anderson EJ, Çakir I, Carrington SJ, Cone RD, Ghamari-Langroudi M, Gillyard T (2016). 60 YEARS OF POMC: Regulation of feeding and energy homeostasis by α-MSH. J Mol Endocrinol.

[165] Blanco EH, Ramos-Molina B, Lindberg I (2015). Revisiting PC1/3 Mutants: Dominant-negative effect of endoplasmic reticulum-retained mutants. Endocrinology.

[166] Baldini G, Phelan KD (2019). The melanocortin pathway and control of appetite-progress and therapeutic implications. J Endocrinol.

[167] Chang JY, Park JH, Park SE, Shon J, Park YJ (2018). The Fat Mass- and Obesity-Associated (FTO) gene to obesity: lessons from mouse models. Obesity.

[168] Hong S, Dimitrov S, Pruitt C, Shaikh F, Beg N (2014). Benefit of physical fitness against inflammation in obesity: Role of beta adrenergic receptors. Brain Behav Immun.

[169] Ochoa MC, Marti A, Azcona C, Chueca M, Oyarza’bal M, Pelach R (2004). Gene–gene interaction between PPAR gamma 2 and ADR beta 3 increases obesity risk in children and adolescents. Int J Obes Relat Metab Disord.

[170] Miranda RC, Vetter SB, Genro JP, Campagnolo PD, Mattevi VS, Vitolo MR (2015). SLC6A14 and 5-HTR2C polymorphisms are associated with food intake and nutritional status in children. Clin Biochem.

[171] Singh RK, Kumar P, Mahalingam K (2017). Molecular genetics of human obesity: A comprehensive review. Comptes Rendus Biologies.

[172] Loos RJF, Burant C, Schur E (2021). Strategies to understand the weight-reduced state: genetics and brain imaging. Obesity.

[173] Marques-Iturria I, Garolera M, Pueyo R, Segura B, Hernan I, Garcia-Garcia I (2014). The interaction effect between BDNF val66met polymorphism and obesity on executive functions and frontal structure. Am J Med Genet B Neuropsychiatr Genet.

[174] Beyer F, Zhang R, Scholz M, Wirkner K, Loeffler M, Stumvoll M (2021). Higher BMI, but not obesity-related genetic polymorphisms, correlates with lower structural connectivity of the reward network in a population-based study. Int J Obes.

[175] Volkow ND, Wise RA (2005). How can drug addiction help us understand obesity?. Nat Neurosci.

[176] Volkow ND, Wise RA, Baler R (2017). The dopamine motive system: implications for drug and food addiction. Nat Rev Neurosci.

[177] Steward T, Miranda-Olivos R, Soriano-Mas C, Fernandez-Aranda F (2019). Neuroendocrinological mechanisms underlying impulsive and compulsive behaviors in obesity: a narrative review of fMRI studies. Rev Endocr Metab Disord.

[178] Moreno-Navarrete JM, Blasco G, Puig J, Biarnes C, Rivero M, Gich J (2017). Neuroinflammation in obesity: circulating lipopolysaccharide-binding protein associates with brain structure and cognitive performance. Int J Obes.

[179] Wang JL, Yang Q, Hajnal A, Rogers AM (2016). A pilot functional MRI study in Roux-en-Y gastric bypass patients to study alteration in taste functions after surgery. Surg Endosc.

[180] Holsen LM, Davidson P, Cerit H, Hye T, Moondra P, Haimovici F (2018). Neural predictors of 12-month weight loss outcomes following bariatric surgery. Int J Obes.

[181] Baboumian S, Pantazatos SP, Kothari S, McGinty J, Holst J, Geliebter A (2019). Functional Magnetic Resonance Imaging (fMRI) of neural responses to visual and auditory food stimuli pre and post Roux-en-Y Gastric Bypass (RYGB) and Sleeve Gastrectomy (SG). Neuroscience.

[182] Zoon H, de Bruijn S, Jager G, Smeets P, de Graaf C, Janssen I (2018). Altered neural inhibition responses to food cues after Roux-en-Y Gastric Bypass. Biol Psychol.

[183] Gu Y, Li G, Wang J, von Deneen KM, Wu K, Yang Y (2020). Comparing the impact of laparoscopic sleeve gastrectomy and gastric cancer surgery on resting-state brain activity and functional connectivity. Front Neurosci.

[184] Wiemerslage L, Zhou W, Olivo G, Stark J, Hogenkamp PS, Larsson EM (2017). A resting-state fMRI study of obese females between pre- and postprandial states before and after bariatric surgery. Eur J Neurosci.

[185] Zeighami Y, Iceta S, Dadar M, Pelletier M, Nadeau M, Biertho L (2021). Spontaneous neural activity changes after bariatric surgery: A resting-state fMRI study. Neuroimage.

[186] Wang J, Li G, Hu Y, Zhang W, Zhang L, Tan Z (2022). Habenular and mediodorsal thalamic connectivity predict persistent weight loss after laparoscopic sleeve gastrectomy. Obesity.

[187] Cerit H, Davidson P, Hye T, Moondra P, Haimovici F, Sogg S (2019). Resting-state brain connectivity predicts weight loss and cognitive control of eating behavior after vertical sleeve gastrectomy. Obesity.

[188] Heinrichs HS, Beyer F, Medawar E, Prehn K, Ordemann J, Floel A (2021). Effects of bariatric surgery on functional connectivity of the reward and default mode network: A pre-registered analysis. Hum Brain Mapp.

[189] Li G, Ji G, Hu Y, Xu M, Jin Q, Liu L (2018). Bariatric surgery in obese patients reduced resting connectivity of brain regions involved with self-referential processing. Hum Brain Mapp.

[190] Li P, Shan H, Liang S, Nie B, Liu H, Duan S (2018). Sleeve gastrectomy recovering disordered brain function in subjects with obesity: a longitudinal fMRI Study. Obes Surg.

[191] Li P, Shan H, Nie B, Liu H, Dong G, Guo Y (2019). Sleeve gastrectomy rescuing the altered functional connectivity of lateral but not medial hypothalamus in subjects with obesity. Obes Surg.

[192] Olivo G, Zhou W, Sundbom M, Zhukovsky C, Hogenkamp P, Nikontovic L (2017). Resting-state brain connectivity changes in obese women after Roux-en-Y gastric bypass surgery: A longitudinal study. Sci Rep.

[193] Zhang W, Ji G, Manza P, Li G, Hu Y, Wang J (2021). Connectome-based prediction of optimal weight loss six months after bariatric surgery. Cereb Cortex.

[194] Wang Y, Ji G, Hu Y, Li G, Ding Y, Hu C (2020). Laparoscopic sleeve gastrectomy induces sustained changes in gray and white matter brain volumes and resting functional connectivity in obese patients. Surg Obes Relat Dis.

[195] Liu L, Ji G, Li G, Hu Y, Jin Q, Hu C (2019). Structural changes in brain regions involved in executive-control and self-referential processing after sleeve gastrectomy in obese patients. Brain Imaging Behav.

[196] Li H, Hu Y, Li G, Zhang W, Wang J, Tan Z (2022). Long-term changes in insula-mesolimbic structural and functional connectivity in obese patients after laparoscopic sleeve gastrectomy. Clin Auton Res.

[197] Bohon C, Garcia LC, Morton JM (2018). Changes in cerebral cortical thickness related to weight loss following bariatric surgery. Obes Surg.

[198] Bohon C, Geliebter A (2019). Change in brain volume and cortical thickness after behavioral and surgical weight loss intervention. Neuroimage Clin.

[199] Michaud A, Dadar M, Pelletier M, Zeighami Y, Garcia-Garcia I, Iceta S (2020). Neuroanatomical changes in white and grey matter after sleeve gastrectomy. Neuroimage.

[200] Rullmann M, Preusser S, Poppitz S, Heba S, Hoyer J, Schutz T (2018). Gastric-bypass surgery induced widespread neural plasticity of the obese human brain. Neuroimage.

[201] Hu Y, Ji G, Li G, Zhang W, Wang J, Lv G (2020). Laparoscopic sleeve gastrectomy improves brain connectivity in obese patients. J Neurol.

[202] Nota MHC, Vreeken D, Wiesmann M, Aarts EO, Hazebroek EJ, Kiliaan AJ (2020). Obesity affects brain structure and function- rescue by bariatric surgery?. Neurosci Biobehav Rev.

[203] Hermann P, Gal V, Kobor I, Kirwan CB, Kovacs P, Kitka T (2019). Efficacy of weight loss intervention can be predicted based on early alterations of fMRI food cue reactivity in the striatum. Neuroimage Clin.

[204] Drummen M, Dorenbos E, Vreugdenhil A, Stratton G, Raben A, Westerterp-Plantenga MS (2018). Associations of brain reactivity to food cues with weight loss, protein intake and dietary restraint during the PREVIEW intervention. Nutrients.

[205] Stillman CM, Jakicic J, Rogers R, Alfini AJ, Smith JC, Watt J (2021). Changes in cerebral perfusion following a 12-month exercise and diet intervention. Psychophysiology.

[206] Legget KT, Wylie KP, Cornier MA, Melanson EL, Paschall CJ, Tregellas JR (2016). Exercise-related changes in between-network connectivity in overweight/obese adults. Physiol Behav.

[207] Mokhtari F, Rejeski WJ, Zhu Y, Wu G, Simpson SL, Burdette JH (2018). Dynamic fMRI networks predict success in a behavioral weight loss program among older adults. Neuroimage.

[208] Levakov G, Kaplan A, Yaskolka MA, Rinott E, Tsaban G, Zelicha H (2021). Neural correlates of future weight loss reveal a possible role for brain-gastric interactions. Neuroimage.

[209] Rodriguez-Ayllon M, Esteban-Cornejo I, Verdejo-Roman J, Muetzel RL, Migueles JH, Mora-Gonzalez J (2020). Physical activity, sedentary behavior, and white matter microstructure in children with overweight or obesity. Med Sci Sports Exerc.

[210] Espeland MA, Erickson K, Neiberg RH, Jakicic JM, Wadden TA, Wing RR (2016). Brain and white matter hyperintensity volumes after 10 years of random assignment to lifestyle intervention. Diabetes Care.

[211] Ten KJ, Veltman DJ, van Bloemendaal L, Barkhof F, Drent ML, Diamant M (2016). Liraglutide reduces CNS activation in response to visual food cues only after short-term treatment in patients with Type 2 Diabetes. Diabetes Care.

[212] Eissele R, Goke R, Willemer S, Harthus HP, Vermeer H, Arnold R (1992). Glucagon-like peptide-1 cells in the gastrointestinal tract and pancreas of rat, pig and man. Eur J Clin Invest.

[213] Kreymann B, Williams G, Ghatei MA, Bloom SR (1987). Glucagon-like peptide-1 7-36: a physiological incretin in man. Lancet.

[214] Vilsboll T, Christensen M, Junker AE, Knop FK, Gluud LL (2012). Effects of glucagon-like peptide-1 receptor agonists on weight loss: systematic review and meta-analyses of randomised controlled trials. BMJ.

[215] Farr OM, Sofopoulos M, Tsoukas MA, Dincer F, Thakkar B, Sahin-Efe A (2016). GLP-1 receptors exist in the parietal cortex, hypothalamus and medulla of human brains and the GLP-1 analogue liraglutide alters brain activity related to highly desirable food cues in individuals with diabetes: a crossover, randomised, placebo-controlled trial. Diabetologia.

[216] Farr OM, Upadhyay J, Rutagengwa C, DiPrisco B, Ranta Z, Adra A (2019). Longer-term liraglutide administration at the highest dose approved for obesity increases reward-related orbitofrontal cortex activation in response to food cues: Implications for plateauing weight loss in response to anti-obesity therapies. Diabetes Obes Metab.

[217] Heni M, Kullmann S, Preissl H, Fritsche A, Haring HU (2015). Impaired insulin action in the human brain: causes and metabolic consequences. Nat Rev Endocrinol.

[218] Kullmann S, Heni M, Fritsche A, Preissl H (2015). Insulin action in the human brain: evidence from neuroimaging studies. J Neuroendocrinol.

[219] Hallschmid M, Benedict C, Schultes B, Fehm HL, Born J, Kern W (2004). Intranasal insulin reduces body fat in men but not in women. Diabetes.

[220] Hallschmid M, Benedict C, Schultes B, Born J, Kern W (2008). Obese men respond to cognitive but not to catabolic brain insulin signaling. Int J Obes.

[221] Kullmann S, Heni M, Veit R, Scheffler K, Machann J, Haring HU (2015). Selective insulin resistance in homeostatic and cognitive control brain areas in overweight and obese adults. Diabetes Care.

[222] Kullmann S, Heni M, Veit R, Scheffler K, Machann J, Haring HU (2017). Intranasal insulin enhances brain functional connectivity mediating the relationship between adiposity and subjective feeling of hunger. Sci Rep.

[223] Heni M, Wagner R, Kullmann S, Gancheva S, Roden M, Peter A (2017). Hypothalamic and striatal insulin action suppresses endogenous glucose production and may stimulate glucose uptake during hyperinsulinemia in lean but not in overweight men. Diabetes.

[224] Kerem L, Hadjikhani N, Holsen L, Lawson EA, Plessow F (2020). Oxytocin reduces the functional connectivity between brain regions involved in eating behavior in men with overweight and obesity. Int J Obes.

[225] van Ruiten CC, Veltman DJ, Nieuwdorp M, IJzerman RG (2022). Brain activation in response to low-calorie food pictures: an explorative analysis of a randomized trial with dapagliflozin and exenatide. Front Endocrinol.

[226] Devoto F, Ferrulli A, Zapparoli L, Massarini S, Banfi G, Paulesu E (2021). Repetitive deep TMS for the reduction of body weight: Bimodal effect on the functional brain connectivity in “diabesity”. Nutr Metab Cardiovasc Dis.

[227] Fatakdawala I, Ayaz H, Safati A, Sakib MN, Hall PA. Effects of prefrontal theta burst stimulation on neuronal activity and subsequent eating behavior: an interleaved rTMS and fNIRS study. Soc Cogn Affect Neurosci. 2021;10.1093/scan/nsab023. 10.1093/scan/nsab023 PMC10074772 33615370

[228] Hare TA, Camerer CF, Rangel A (2009). Self-control in decision-making involves modulation of the vmPFC valuation system. Science.

[229] Karlsson J, Taft C, Ryden A, Sjostrom L, Sullivan M (2007). Ten-year trends in health-related quality of life after surgical and conventional treatment for severe obesity: the SOS intervention study. Int J Obes.

[230] Courcoulas AP, Christian NJ, Belle SH, Berk PD, Flum DR, Garcia L (2013). Weight change and health outcomes at 3 years after bariatric surgery among individuals with severe obesity. JAMA.

[231] King WC, Hinerman AS, Belle SH, Wahed AS, Courcoulas AP (2018). Comparison of the performance of common measures of weight regain after bariatric surgery for association with clinical outcomes. JAMA.

