# Supplementary material for: Brain functional and structural magnetic resonance imaging of obesity and weight loss interventions
Source: Mol Psychiatry. 2023 Mar 14;28(4):1466–79. doi: 10.1038/s41380-023-02025-y (PMC10208984; doi:10.1038/s41380-023-02025-y)
Supplement: Supplementary file 1 — Supplementary Table 1 [file 41380_2023_2025_MOESM1_ESM.doc]

**Supplementary Table 1**. MRI studies related to obesity.

| **Authors (year)/(ref #)** | **Study group** | **Imaging task** | **Main findings** |
| --- | --- | --- | --- |
| **Neural responsivity (fMRI)** | | | |
| Blechert *et al*. (2016)/[13] | N=32 healthy subjects (Age 22.4±2.41, BMI 22.4±2.23) | Passively viewing available and unavailable foods of either high or low-caloric density | - Available compared to unavailable foods elicited higher palatability ratings as well as stronger neural activation in the orbitofrontal cortex (OFC), amygdala, and left caudate nucleus as well as in the anterior cingulate cortex (ACC).  - Availability effects in the caudate were mainly attributable to the high calorie condition (availability × calorie density interaction). |
| Wiemerslage *et al*. (2016)/[30] | N=30 participants homozygous for the rs9939609 SNP: N=17 AA genotype (Age 26±3, BMI 27±4), and N=17 TT genotype (Age 26±2, BMI 24±3) | Cue reactivity with low-calorie food, high-calorie food, and control visual cues | - People with the FTO risk allele genotype (AA) had increased activity compared with the non-risk (TT) genotype in the posterior cingulate, cuneus, precuneus and putamen.  - Higher BMI in the AA genotype was associated with reduced activity to food images in areas important for emotion (cingulate cortex), but also in areas important for impulse control (frontal gyri and lentiform nucleus).  - Behavioral inhibition system as well as subscales of the behavioral activation system correlated with BMI oppositely in each genotype. |
| Rapuano et al. (2016)/[136] | Age 14.4±1.3, range 12-16. N=18 Obese subjects (BMI 33.2±2.51), N=19 healthy weight (BMI 20.15±2.05) | Twelve food and 12 non-food high-resolution commercials were matched for length (mean food commercial = 28.4 s; mean control commercial = 28.9 s) | - Compared with non-food commercials, food commercials more strongly engaged regions involved in attention and saliency detection (occipital lobe, precuneus, superior temporal gyri, and right insula) and in processing rewards [left and right nucleus accumbens (NAc) and left orbitofrontal cortex (OFC)].  - Activity in the left OFC and right insula further correlated with subjects' percent body fat at the time of the scan.  - This reward-related activity to food commercials was accompanied by the additional recruitment of mouth-specific somatosensory-motor cortices. |
| Ulrich *et al*. (2016)/[137] | N=24 (Age 24.6±3.0, BMI 23.6±2.1 kg/m^2^), N=18 (Age 23.5±2.8, BMI 24.0±3.2 kg/m^2^) | fMRI, glucose was intravenously infused in healthy human male participants while seeing images depicting low-caloric food (LC), high-caloric food (HC), and non-food (NF) during a food/NF discrimination task | - Under unmodulated fasting baseline conditions, VTA activation was greater during HC compared with LC food cues.  - Subsequent to infusion of glucose, this difference in VTA activation as a function of caloric load leveled off and even reversed. |
| Demos *et al*. (2017)/[18] | N=30 participants completed 4 nights of 6 hours/night time-in-bed (short sleep) and 4 nights of 9 hours/night (long sleep) in random counterbalanced order (Age 36.7±10.8, BMI range 20.4-40.7) | Cue reactivity with food, and non-food images | - Increased activity to food in short- vs. long-sleep in regions of reward processing (e.g., nucleus accumbens/putamen) and sensory/motor signaling.  - Greater food cue-responsivity during short sleep in an inhibitory control region [right inferior frontal gyrus (IFG)] and ventral medial prefrontal cortex (vMPFC), which has been implicated in reward coding and decision-making. |
| Chin *et al*. (2018)/[17] | N=32 obesity subjects (Age range 19-60, BMI range 30-39.9) | Cue reactivity with food, and non-food objects | - Satiety decreased and thirst increased during the scan.  - While some subjective, self-reported hunger, satiety and related constructs may be moderating fMRI food-cue reactivity (fMRI-FCR), these constructs do not appear to be salient influences on appeal/fMRI-FCR in people with obesity undergoing fMRI. |
| Belfort-DeAguiar *et al.* (2018)/[12] | N=10 obese (BMI 34±3) and N=10 normal-weight (BMI 23±2) nondiabetic healthy adults | Reactivity to food and non-food images during normoglycemia and hyperglycemia. | - In NW individuals, hyperglycemia promotes a brain activity pattern toward a decrease in motivation for food (hypothalamus and caudate) and an increase in reward-motivation (insula and putamen) to neutral (non-food) objects  - OB individuals have a distinct brain activity pattern in response to hyperglycemia, namely, enhanced activity in brain regions regulating reward-motivation (insula and putamen) and self-control (aPFC/dlPFC)  - Obesity may induce a state of augmented desire for food that is insensitive to the peripheral signals concerning the body’s energy storage |
| Gearhardt *et al*. (2020)/[21] | N=193 adolescents (BMI 24.10±5.35) | Cue reactivity with unhealthy and healthier fast food and nonfood commercials | - Greater responses to healthier FF relative to nonfood commercials in regions associated with reward, memory, and sensorimotor processes predicted greater total food and unhealthier food intake, but not healthier food intake.  - Lower activation in neural regions associated with visual attention and salience  to unhealthy relative to healthier FF commercials predicted healthier food intake. |
| Ghobadi-Azbari *et al*. (2022)/[133] | N=50 overweigh/obese participants (Age 21-59, 35.33±9.82, BMI 29.67±3.56 kg/m^2^) | View food and neutral cues during fMRI scanning | - The ventral tegmental area (VTA), amygdalae, and orbitofrontal cortices (OFC) showed significant food cue-reactivity |
| Puzziferri *et al*. (2016)/[26] | N=15 women with severe obesity (Age 40.6±12, BMI range 35-50), and 15 age-matched lean women (BMI range 18.5-24.9) | Food task with high-calorie savory foods, high-calorie sweet foods, and low-calorie foods, which performed before and after standard meal intake | - When fasting, brain perfusion did not differ significantly between groups; and both groups showed significantly increased activity in the neo- and limbic cortices and midbrain compared with baseline. Once fed, the lean group showed significantly decreased activation in these areas, especially the limbic cortex, whereas the group with severe obesity showed no such decreases.  - After eating, appeal ratings of food decreased only in lean women.  - Within groups, hunger decreased and fullness increased fasted to fed. |
| Carnell *et al*. (2017)/[16] | N=10 obese/overweight individuals (Age 15.8±1.8, BMI 32.6±7.1), 16 lean with obese/overweight mothers (lean-HR, Age 15.5±1.4, BMI 20.9±2.2), and 10 lean with lean mothers (lean-LR, Age 16.0±1.9, BMI 21.0±1.7) | Neural responses to words denoting high or low energy density (ED) foods and non-foods | - Food compared with non-food words activated a distributed emotion/reward system including insula and pregenual anterior cingulate cortex (pgACC).  - Participants who were at increasing risk for obesity exhibited progressively weaker activation of an attentional/regulatory system including dorsolateral prefrontal cortex (PFC), dorsal ACC, and head of caudate. These group differences were most apparent for neural responses to high-compared with low-ED foods.  - Lean-HR (compared with lean-LR and overweight) adolescents reported greater desire for high-ED foods.  - Meal intake was greatest for the overweight, then lean-HR, then lean-LR groups. |
| Masterson *et al*. (2019)/[25] | N=41, 7-9 years old  children (25 healthy weight, 16 with overweight/obesity) | Food stimuli with low- and high-energy dense food images following exposure to either food or toy commercials. | - Meal intake did not differ by commercial condition  - Relative to toy commercials, food commercials reduced brain response to high-energy food stimuli in cognitive control regions, including bilateral superior temporal gyri, middle temporal gyrus, and inferior frontal gyrus  - Children with overweight/obesity showed increased response in orbitofrontal cortex, fusiform gyrus, and supramarginal gyrus to high-energy stimuli following food commercials.  - Food commercial exposure affected children’s subsequent processing of food cues by reducing engagement of the prefrontal cortex |
| Dodd *et al*. (2020)/[19] | N=16 college students (BMI 25.99±5.44) | Cue reactivity with three visual information: (1) food/drink image; (2) food/drink words; (3) food/drink image and words. | - Compared to a written dietary record, that the visualization of personal images of diet evoked greater brain activation in memory regions (e.g., superior frontal gyrus) along with mediating emotion (e.g., thalamus, putamen, anterior cingulate cortex), imagery and executive functions (e.g., inferior orbitofrontal gyrus, fusiform, and parietal lobe). |
| Veit *et al*. (2020)/[29] | N=9 overweight/obese subjects (BMI 30.38±2.93) and N=9 normal weight controls (BMI 21.78±1.25) | Cue reactivity with 10 different meals in 10 different portion sizes | - Participants of all weight groups enhanced activation of the self-control network and reduced their portion size when adopting a health mindset.  - Under the pleasure mindset, persons with overweight and obesity showed heightened activity in parts of the taste cortex, while the fullness mindset caused reduced activation in the ventral striatum. |
| Bogdanov *et al*.  (2020)/[14] | N=15 severely obese subjects (BMI 41.2±2.1) and N=15 normal weight controls (BMI 21.8±2.6) | Cue reactivity with guessing-task paradigm | - Greater reward-related activations were present in the dorsolateral prefrontal cortex, and precuneus/posterior cingulate of obese subjects compared to controls.  - Obese subjects exhibited longer choice times after repeated reward and lower circulating ghrelin levels than lean controls.  - Reduced ghrelin levels significantly predicted slower post-reward choices and reward-related hyperactivity in dorsolateral prefrontal cortices in obese subjects. |
| Stopyra *et al*.  (2021)/[28] | N=25 obese females (BMI 35.57±3.88) and 25 normal-weight females (BMI 21.77±1.48) | Cue reactivity with high-calorie food and nonfood image stimulations in viewing condition and distraction condition after receiving either water or glucose directly into the stomach using a nasogastric tube. | - Food craving regulation was associated with increased activation in fronto-parietal regions in participants with obesity when compared to healthy controls.  - The reduction of food craving was related to increased activation in the  lingual gyrus in individuals with obesity following the infusion of water. |
| Jastreboff *et al*. (2016)/[139] | N=24 obese subjects (Age 15.3±1.8, BMI 34.4±4.7) and 14 lean subjects (Age 15.8±1.6, BMI 21.8±2.3) | Baseline resting state scans, and post-glucose/fructose drink resting and perfusion scans were acquired in an alternating fashion | - In response to drinking glucose, obese adolescents exhibited decreased brain perfusion in brain regions involved in executive function (prefrontal cortex, PFC) and increased perfusion in homeostatic appetite regions of the brain (hypothalamus). Conversely, lean adolescents demonstrated increased PFC brain perfusion and no change in perfusion in the hypothalamus.  - Obese adolescents demonstrated attenuated suppression of serum acyl-ghrelin and increased circulating insulin level following glucose ingestion; the change in acyl-ghrelin and insulin levels following both glucose and fructose ingestion was associated with increased hypothalamic, thalamic, and hippocampal blood flow in obese relative to lean adolescents.  - In all subjects there was greater perfusion in the ventral striatum with fructose relative to glucose ingestion.  - Reduced connectivity between executive, homeostatic, and hedonic brain regions was observed in obese adolescents. |
| Geha *et al*. (2017)/[22] | Two independent fMRI data sets: participants tasted milkshake (n=15 healthy weight and 15 obese), and the second with participants at rest (n=33 healthy weight and 28 obese). | Taste stimulus with milkshake or a tasteless solution | - In the resting state and during milkshake consumption global brain connectivity (GBC) is consistently decreased in the ventromedial and ventrolateral prefrontal cortex, insula and caudate nucleus, and increased in brain regions belonging to the dorsal attention network including premotor areas, superior parietal lobule, and visual cortex.  - During milkshake consumption, but not at rest, additional decreases in GBC are observed in feeding-related circuitry including the insula, amygdala, anterior hippocampus, hypothalamus, midbrain, brainstem and somatomotor cortex.  - GBC in the fusiform gyrus differed depending on BMI group and condition.  - Greater connectivity in the obese was observed during milkshake consumption and lower connectivity at rest. |
| Winter *et al*. (2017)/[31] | N=162 healthy-weight adolescents (BMI 20.82±1.93) at baseline, and were assessed annually over a 3-y follow-up | Taste stimulus with chocolate milkshake | - Elevated activation in the medial prefrontal cortex and supplementary motor area, cingulate gyrus, cuneus and occipital gyrus, and insula in response to milkshake receipt predicted greater weight variability.  - Greater activation in the precuneus and middle temporal gyrus predicted lower weight variability. |
| Bohon *et al*. (2017)/[15] | N=8 overweight (BMI > 85th %ile), and 10 healthy weight (BMI between 5th and 85th %ile) | Cue reactivity with milkshake, water image and/or taste stimulus | - Greater response to milkshake taste receipt in overweight children in the right insula, operculum, precentral gyrus, and angular gyrus, and bilateral precuneus and posterior cingulate.  - No group differences were found for brain response to a visual food cue.  - Exploratory analyses revealed interactions between self-report measures of eating behavior and weight status on brain response to taste. |
| Shearrer *et al*. (2018)/[27] | N=108 healthy-weight adolescents: N=53 who were at high risk by virtue of parental obesity status; N=55 who were low risk | Food stimuli included 4 milkshakes, which systematically varied in sugar and fat content, a calorie-free tasteless solution, and images of appetizing foods and glasses of water | - High-risk compared with low-risk adolescents showed greater caudate, gustatory, and oral somatosensory responses to the high-sugar milkshake than to the tasteless solution.  - Parental weight status is associated with greater striatal, gustatory, and somatosensory responses to high-sugar food in their adolescent offspring, which theoretically contributes to an increased risk of future overeating. |
| Ebrahimi *et al.* (2019)/[20] | N=62 healthy participants in the appetitive conditioning session (day 1), and a subsample (n = 33) further completed extinction (day 2) and a reinstatement test (day 3). | An appetitive conditioning paradigm with liquid food rewards in combination with a 3-day design | - A return of conditioned responding during the reinstatement test, evident by enhanced skin conductance responses, accompanied by enhanced BOLD responses in the amygdala.  - Psychophysiological reinstatement intensity was significantly anticorrelated with ventromedial prefrontal cortex (vmPFC) activation, and marginally anticorrelated with enhanced amygdala-vmPFC connectivity during late reinstatement. |
| Sadler *et al*. (2021)/[138] | N=154 healthy weight adolescents (Age range 14-17, BMI range 18-25 kg/m^2^). | Using functional MRI to examine how brain response to a palatable taste and proceeding cues changed over repeated exposures. | - Caudate and posterior cingulate cortex (PCC) response increased with repeated cue presentations  - Oral somatosensory cortex and insula response increased with repeated milkshake tastes  - Adolescents with familial obesity risk showed higher cue-evoked caudate response across time, compared to the low risk group  - Reward-responsiveness positively correlated with right oral somatosensory cortex/insula response to milkshake over time |
| Jacobson *et al*. (2019)/[24] | N=40 adults (BMI 26.3±0.6) | Cue-reactivity task with olfactory stimulation under hunger and satiety condition | - Activation in reward areas (caudate, putamen, lentiform nucleus), frontal regions and sensory area (insula) decreased as BMI increased.  - A greater BMI is associated with an increased activation in the primary olfactory (the piriform cortex)) and odor memory areas (entorhinal cortex), which was observed during a hunger state. |
| Han *et al.*  (2021)/[23] | N=17 participants with obesity (BMI 38.2±4.5) and 21 participants with normal weight (BMI 21.8±2.1) | Chocolate (high-energy-dense food) and cucumber (low-energy-dense food) odor stimuli | - Compared with normal-weight controls, participants with obesity had lower odor sensitivity (phenylethyl alcohol) and decreased odor discrimination ability.  - Participants with obesity demonstrated greater brain activation in response to chocolate compared with cucumber odors in the bilateral inferior frontal operculum and cerebellar vermis, right ventral anterior insula extending to putamen, right middle temporal gyrus, and right supramarginal areas. |
| Janssen *et al*. (2017)/[35] | N=76 healthy subjects (Age range 18-53, BMI range 19-35) | A food Stroop task with different color of the words (high-calorie food, positively valanced emotional, and neutral words) | - Increased obesity scores were associated with diminished lateral prefrontal cortex responses during food attentional bias.  - This was accompanied by decreased goal-directed control of food choices following outcome devaluation. |
| Verdejo-Román *et al*. (2017)/[37] | The sample comprised 21 adults with obesity (BMI>30), 21 with overweight (BMI range 25-30), and 39 with normal weight (BMI<25) | Two tasks that involve the processing of food (Willing to Pay) and monetary rewards (Monetary Incentive Delay) | - Obesity is associated with greater food-evoked responsivity in the ventral and dorsal striatum, and overweight is associated with greater monetary-evoked responsivity in the ventral striatum, the amygdala, and the medial frontal cortex.  - Food and monetary-feedback evoked neural activations showed a linear positive relationship with BMI, whereas monetary reward-anticipation evoked neural activations showed an inverted U-shape association with BMI. That is, individuals with BMIs between 27 and 32 had greater responsivity to monetary stimuli. |
| Verdejo-Román *et al*. (2017)/[38] | N=39 excess weight individuals (Age 33.59±6.23, BMI 30.41±3.69), and 37 normal weight individuals (Age 33.00±6.53, BMI 22.28±1.77) | Willingness to Pay for Food task and the Monetary Incentive Delay task | - Excess weight was associated with decreased functional connectivity during the processing of food rewards in a network involving primarily frontal and striatal areas, and increased functional connectivity during the processing of monetary rewards in a network involving principally frontal and parietal areas.  - The processing of food and monetary rewards involve segregated neural networks, and both are altered in individuals with excess weight. |
| Merchant *et al*. (2020)/[36] | N =93 early middle-aged and higher BMI participants (BMI 31.41±3.91) | Willingness-to-pay task that quantifies trial-by-trial valuation of 30 healthy and 30 unhealthy food items. | - There were robust positive linear relationships between self-reported value of food items and the corresponding BOLD response in the vmPFC, anterior VS, bilateral anterior insula (AI), and the ACC when making decisions about snack food items.  - No relationship between valuation and BMI nor Healthy Eating Index (HEI), and HEI was inversely related to subjective valuation of unhealthy foods.  - Higher BMI individuals do not display biases in the neurocognitive substrates of subjective valuation, and that the general functioning of this system may not relate to BMI or healthy eating. |
| Contreras-Rodriguez *et al*. (2020)/[39] | N=20 obese subjects (BMI 33.36±2.58), N=19 overweight subjects (BMI 27.60±1.51) and N=33 normal weight controls (BMI 22.23±1.72) | Cue reactivity with the willingness to pay task for functional versus standard foods | - Individuals with OB, relative to HW, showed more similar willingness to pay for functional and standard food.  - Hyperactivation in the ventral posterior cingulate cortex (vPCC), and the right angular gyrus, as well as an increased functional connectivity between the vPCC.  - The activation in the vPCC showed a linear positive relationship with BMI. |
| Spetter *et al*. (2018)/[41] | N=15 Fifteen fasted normal-weight, young men received intranasal oxytocin or placebo | Food stimuli and a monetary incentive delay task (MID). | - Oxytocin compared to placebo increased activity in the ventromedial prefrontal cortex, supplementary motor area, anterior cingulate, and ventrolateral prefrontal cortices in response to high- vs. low-calorie food images in the fasted state and reduced calorie intake by 12%.  - During anticipation of monetary rewards, oxytocin compared to placebo augmented striatal, orbitofrontal and insular activity without altering MID performance  - During the anticipation of generalized rewards, oxytocin stimulates dopaminergic reward-processing circuits. In contrast, oxytocin restrains food intake by enhancing the activity of brain regions that exert cognitive control |
| Kube et al. (2018)/[34] | N=19 individuals with obesity (BMI > = 30, 10 female) and N=23 lean control participants  (BMI 18.5–24.9, 11 female) | Probabilistic learning task during  fMRI, in which they learned to choose in separate monetary gain, loss, and neutral conditions. | - Obese patients made a significantly lower number of correct choices and accumulated a significantly lower overall monetary outcome than lean control participants  - Obese patients showed higher medial prefrontal cortex responses to monetary losses  - No significant group differences in prediction errors (PE) related activity  - Increased functional connectivity between the ventral striatum and insula in individuals with obesity  - Obesity is associated with aberrant value representations for monetary losses, alterations in functional connectivity during the processing of learning outcomes, as well as a decreased reinforcement-based learning performance |
| Cheke *et al*. (2017)/[44] | N=16 obese individuals (Age 27.7±5.7, BMI 34.29±4.0), and 16 lean individuals (Age 27.3±5.9, BMI 21.29±2.1) | What-Where-When episodic memory test (the “Treasure-Hunt Task”) | - In lean participants, the Treasure-Hunt task elicited significant activity in regions of the brain known to be important for recollecting episodic memories, such as the hippocampus, angular gyrus, and dorsolateral prefrontal cortex.  - Both obesity and insulin resistance were associated with significantly reduced functional activity throughout the core recollection network. |
| Weygandt *et al*. (2019)/[42] | N=30 obese patients (BMI 34.4±3.1) | - Cue-reactivity task with neutral objects and high-calorie foods images, and a food-specific delay discounting task  - Participants were scanned preceding the twelve-week diet (T-3), immediately thereafter (T0), and 12 (T12), 24 (T24) and 36 months (T36) after the diet | - Dietary success (ΔBMI) was negatively associated with Pavlovian activity primarily in left hippocampus and positively associated to goal-directed activity primarily in right inferior parietal gyrus  - There was a strong negative association between interactions of stable Pavlovian (visual areas) and goal-directed DM signals (right insular) and the ΔBMI |
| Morys *et al*. (2018)/[32] | N=30 lean (BMI 22.14±1.81) and N=26 obese subjects (BMI 34.32±3.37) | A primed delay discounting paradigm using gustatory and visual cues of positive, neutral and negative valence | - Obese subjects were more susceptible to priming with negative gustatory cues towards delayed choices as opposed to lean subjects. This was related to lower activity in the left dorsolateral prefrontal cortex during priming  - Modulation of functional connectivity between the DLPFC and the vmPFC by the priming effect correlated negatively with BMI. |
| Miranda-Olivos *et al*. (2021)/[141] | N=35 obese patients (N=10 BED and N=25 non-BED) and N=31 lean controls | Delay discounting task during fMRI | - Increased discounting rates were associated with decreased activity in the left anterior insula in participants with obesity compared to controls when choosing immediate rewards over delayed rewards  - No significant difference between BED subsample to the other groups |
| Zhang *et al.* (2022)/[43] | N=30 obese subjects (Age 30.86±1.54, BMI 35.97±0.87) and 30 normal-weight subjects (Age 28.60±1.17, BMI 21.12±0.40) | Delay discounting task with choice and feedback stages | - During decision-making in the delay discounting task, obese subjects compared with normal weight subjects had greater activation in the dorsolateral prefrontal cortex (DLPFC) and posterior parietal cortex, which was associated with greater discounting rate and weaker cognitive control.  - The association between DLPFC activation and cognitive control was mediated by discounting rate.  - There were decreased connectivity of DLPFC–inferior parietal cortex and angular gyrus–caudate in obese subjects. |
| Hsu *et al*. (2017)/[33] | N=20 females with obesity and sweet food addiction (O & SFA, Age 25.20±3.16, BMI 33.09±3.01), and 20 controls (Age 23.75±2.86, BMI 21.58±2.92) | Go/No-go task | - The O & SFA group exhibited a higher score for impulsivity, and lower brain activation when processing response inhibition over the right Rolandic operculum and thalamus than controls.  - Both O & SFA and control groups exhibited activation of the insula and caudate during error processing. The activation over the left insula, precuneus, and bilateral putamen were higher in the subjects with O & SFA than for those in the control group. |
| **Resting-State fMRI** | | | |
| Moreno-Lopez *et al*. (2016)/[53] | N= 60 adolescents with excess weight (Age 14.67±1.70, BMI 29.26±3.84) and 55 normal weight controls (Age 15.11±1.82, BMI 20.84±2.39) | Resting state scans | - Adolescent obesity display reduced global functional connectivity in the insula/operculum, the middle temporal cortex and the dorsolateral prefrontal cortex (DLPFC).  - Adolescent obesity show reduced regional seed-based connectivity between the insula and the dorsal anterior cingulate cortex, and between the middle temporal cortex and the posterior cingulate cortex and the cuneus/precuneus; and increased connectivity between the insula/operculum and the cuneus, between the middle temporal cortex and the orbitofrontal cortex, and between the DLPFC seed and the primary visual cortex, these networks were correlated with sensitivity to reward. |
| Park *et al*. (2016)/[56] | N=41 healthy weight (HW) group (Age 29.83±9.95, BMI 22.03±1.67), and 41 non-HW group (Age 33.24±10.09, BMI 30.88±3.82) | Resting state scans | - Frontoparietal and cerebellum networks showed group-wise differences between HW and non-HW groups.  - Frontoparietal network showed a high correlation with TFEQ disinhibition scores. Both frontoparietal and cerebellum networks showed a high correlation with body mass index (BMI) scores. |
| Nakamura *et al*. (2017)/[54] | N=185 subjects (Age 37.4±19.4, BMI 26.1±5.48) | NKI-Rockland resting state fMRI dataset | - Caudate-precuneus functional connectivity showed inverse correlation to the underlying tendency of obesity prevention. |
| Contreras-Rodríguez *et al*. (2017)/[48] | N=42 excess weight participants (Age 33.59±6.16, BMI 30.51±3.63), and 39 normal weight participants (Age 33.07±6.73, BMI 22.09±1.74) | Resting state scans, 12 weeks follow-up | - Participants with excess weight displayed increased functional connectivity between the ventral striatum and the medial prefrontal and parietal cortices, and between the dorsal striatum and the somatosensory cortex.  - Dorsal striatum connectivity correlated with food craving, and predicted BMI gains. |
| Beyer *et al*. (2017)/[47] | N=712 old participants of the Leipzig Research Center for Civilization Diseases cohort (Age range 60-80, BMI 27.6±4.2). | Resting state scans | - A higher BMI was significantly associated with lower default mode functional connectivity in the posterior cingulate cortex and precuneus.  - Lower functional connectivity in BMI-associated areas correlated with worse executive function.  - Higher BMI correlated with stronger head motion. |
| Baek *et al*. (2017)/[46] | N=20 obese subjects (BMI 33.4±3.9), binge eating disorder patient (BMI 33.0±2.4), and 40 healthy weight controls (BMI 22.5±2.0) | Resting state scans | - Obese subjects exhibited significantly reduced global and local network efficiency as well as decreased modularity compared with healthy controls, showing disruption in small-world and modular network structures.  - In regional metrics, the putamen, pallidum and thalamus exhibited significantly decreased nodal degree and efficiency in obese subjects.  - Obese subjects also showed decreased connectivity of cortico-striatal/cortico-thalamic networks associated with putaminal and cortical motor regions. |
| Meng *et al*. (2018)/[52] | N=26 obese patients and N=28 normal weight subjects | Resting-state fMRI | - Obese patients showed significantly increased shortest path length (Lp) and decreased global efficiency (Eglob).  - Obese patients showed decreased nodal-degree/efficiency in frontal (mOFC, rACC), striatal (caudate/NAc) and limbic regions (insula, amygdala, hippocampus/parahippocampal gyrus) and thalamus  - Obese patients showed decreased functional connectivity in a sub-network associated with the right rACC  - Lp and Eglob were negatively correlated with BMI, and BMI was negatively correlated with nodal-degree/efficiency of the mOFC |
| Gupta *et al*. (2018)/[50] | N=43 women and N=43 men | 10-minute resting functional magnetic resonance imaging scan | - In both men and women, increased BMI was associated with increased slow-5 activity in the left globus pallidus (GP) and substantia nigra  - In women only, increased BMI was associated with increased slow-4 activity in the right GP and bilateral putamen  - In women, increased BMI was associated with reduced slow-5 connectivity between the left GP and putamen and the emotion and cortical regulation regions, but in men, increased BMI was associated with increased connectivity with the medial frontal cortex.  - In both men and women, increased BMI was associated with increased slow-4 connectivity between the right GP and bilateral putamen and the emotion regulation and sensorimotor-related regions.  - The stronger relationship between increased BMI and decreased connectivity of core reward network components with cortical and emotion regulation regions in women may be related to the greater prevalence of emotional eating |
| Rashid *et al*. (2019)/[57] | N=27 participants with metabolic syndrome (Mets), N=26 participants with pre-Mets and N=25 healthy controls | Resting-state fMRI | - Participants with MetS demonstrated reduced positive connectivity between the DMN seed and left superior frontal regions, and reduced negative connectivity between the DMN seed (isthmus of the cingulate) and left superior parietal, left postcentral, right precentral, right superior temporal and right superior parietal regions  - MetS is associated with alterations in FC between the DMN and other regions of the brain |
| Zhang *et al*. (2019)/[61] | N=28 obese (BMI 37.99±6.48) and N=28 normal-weight female controls (BMI 21.35±1.37) | Resting-state fMRI | - Compared with normal-weight controls, obese females showed an increased degree centrality in the left ventral striatum/caudate and decreased degree centrality in right orbitofrontal cortex  - Obese females showed directional effective connectivity between left ventral striatum/caudate and several regions (left inferior temporal gyrus, fusiform gyrus, postcentral gyrus, and right precentral gyrus)  - Obese females showed directional effective connectivity between the right OFC and several regions (left middle temporal gyrus, cuneus, OFC, superior temporal gyrus, middle frontal gyrus, and right inferior parietal lobule) |
| Shapiro *et al*. (2019)/[59] | N=18 typically developing children (age: 5.8±0.5) | Resting-state fMRI | - The kilocalories from the eating in the absence of hunger (EAH kcal) was positively associated with activity of the nucleus accumbens.  - EAH kcal was negatively associated with intrinsic prefrontal cortex connectivity to the striatum |
| Park *et al*. (2020)/[55] | UKB: N=1007 non-healthy weight subjects (BMI 29.61±3.97) and N=490 healthy weight subjects (BMI 22.82±1.65)  HCP: N=291 non-healthy weight subjects (BMI 28.45±2.85) and N=296 healthy weight subjects (BMI 22.31±1.78)  eNKI-RS: N=276 non-healthy weight subjects (BMI 30.65±4.83)  SVH: N=27 non-healthy weight subjects (BMI 28.19±2.66) and N=3 healthy weight subjects (BMI 23.77±0.74) | Resting state scans | - Brain regions containing the reward circuit positively associated with obesity phenotypes, while brain regions for sensory processing showed negative associations. |
| Zhang *et al*. (2020)/[63] | N=23 obese males (BMI 39.13±4.81) and N=23 normal-weight males (BMI 21.56±1.63) | Resting state scans | - Obese group showed increased amplitude of low-frequency values in left fusiform gyrus/amygdala, inferior temporal gyrus, hippocampus/parahippocampal gyrus, and bilateral caudate but decreased values in right superior temporal gyrus.  - Increased FC between left caudate and right superior temporal gyrus, left fusiform gyrus/amygdala and left inferior temporal gyrus, right caudate and left fusiform gyrus/amygdala, and right caudate and left hippocampus/parahippocampal gyrus.  - Dutch Eating Behavior Questionnaire-Emotional scores were positively correlated with FC between left hippocampus/parahippocampal gyrus and right caudate but negatively correlated with FC between left fusiform gyrus/amygdala and left inferior temporal gyrus. |
| Ding *et al*. (2020)/[49] | N=35 obese males (BMI 39.24±0.88) and N=35 normal-weight males (BMI 20.84±0.36) | Resting state scans | - Compared with NW, OB showed reduced FC strength in the ventromedial prefrontal cortex and posterior cingulate cortex/precuneus within the default-mode network, dorsal anterior cingulate cortex within the salience network (SN), bilateral dorsolateral prefrontal cortex-angular gyrus within the frontoparietal network (FPN), and increased FC strength in the insula (INS).  - The dorsal anterior cingulate cortex FC strength was negatively correlated with craving for food cues, left dorsolateral prefrontal cortex FC strength was negatively correlated with Yale Food Addiction Scale scores, and right INS FC strength was positively correlated with craving for high-calorie food cues.  - Compared with NW, OB also showed increased FC between the SN and FPN driven by altered FC of bilateral INS and anterior cingulate cortex-angular gyrus. |
| Zhang *et al*. (2021)/[62] | N=24 obese males (BMI 38.58±5.04) and N=24 normal-weight males (BMI 22.01±1.77) | Resting state scans | - Decreased degree centrality was observed in left ventral medial prefrontal cortex (vmPFC) and right parahippocampal/hippocampal gyrus in group with obesity.  - The group with obesity demonstrated increased effective connectivity between left vmPFC and several regions (left inferior temporal gyrus, left supplementary motor area, right insular cortex, right postcentral gyrus, right paracentral lobule and bilateral fusiform gyrus). |
| Tan *et al*. (2021)/[60] | N=56 obese subjects (BMI 36.68±0.79) and 46 normal weight subjects (BMI 21.13±0.48) | Resting-state fMRI data | - OB compared with NW had more occurrences and a longer mean dwell time a state where the basal ganglia network had positive FC with other networks, and obese subjects also had higher FC of basal ganglia-salience network in other states.  - Body mass index was positively correlated with mean dwell time and FCs of basal ganglia-anterior default mode network and basal ganglia-salience network. |
| Legget *et al.*  (2021)/[51] | N=24 obesity-prone individuals (BMI 26.2±3.1) and 25 obesity-resistant individuals (BMI 20.8±2.1) | Resting-state fMRI data was performed in the fasted state during 10 min of rest (300 image volumes), during which participants rested with eyes closed. | - Greater between-network connectivity was observed in the basal ganglia and right dorsolateral prefrontal cortex in obesity-prone individuals, driven by stronger associations with lateral sensorimotor and inferior visual networks, respectively.  - Between-network connectivity in the basal ganglia was significantly correlated  with hunger AUC and satiety AUC. |
| Ravichandran *et al.*  (2021)/[58] | N=150 obese/overweight participants (Ages range 18–55, 47 male and 103 female) | Resting-state scans were acquired with eyes closed and scan duration range 8 min 6 s–10 min 6 s. | - Individuals with food addiction had greater connectivity between brainstem regions and the orbital frontal gyrus compared to individuals with no food addiction.  - Females with food addiction had greater connectivity in the salience and emotional regulation networks and lowered connectivity between the default mode network and central executive network compared to males with food addiction.  - Increased connectivity between regions of the reward network was positively associated with scores on the General Food Cravings Questionnaire-Trait |
| Zhao *et al.* (2022)/[45] | N=20 normal-weight undergraduates (Age 18.3±0.8), and 18 overweight/obese undergraduates (Age 18.5±1.86) | The resting-state fMRI data were acquired 8 min continuously, resulting in 240 functional volumes. | - The regional homogeneity of right angular gyrus was smaller in obese undergraduates than that in normal-weight undergraduates.  - Functional connectivity of the left middle temporal cortex and the right angular gyrus were found to be smaller in obese group than that in normal-weight group by setting the right angular gyrus as seed region. |
| Hogenkamp *et al*. (2016)/[146] | N=17 obese females (Age 39±11, BMI 42.3±4.8) and 12 normal weight females (Age 36±12, BMI 22.7±1.8) | Resting state scans performed before and after consumption of a standardized meal | - Obese females had increased low-frequency activity in clusters located in the putamen, claustrum and insula.  - No changes in group differences after food intake.  - Self-reported hunger dropped and plasma glucose concentrations increased after food intake; these changes did not differ between the BMI groups. |
| Avery *et al*. (2017)/[145] | N=52 obese participants (Age 37.3±8.0, BM 35.3±3.6), and 18 healthy weight participants (Age 35.1±10.5, BMI 21.5±1.5) | Resting state scans during varying interoceptive states, both while fasting and after a standardized meal | - Obese and healthy weight individuals exhibited opposing patterns of eating-related functional connectivity between the dorsal mid-insula and multiple brain regions involved in reward, valuation, and satiety.  - Healthy weight participants exhibited a significant positive relationship between changes in hunger and changes in medial orbitofrontal functional connectivity, while obese participants exhibited a complementary negative relationship between hunger and ventral striatum connectivity to the mid-insula. |
| Voigt *et al.* (2021)/[144] | N=41 participants including 14 obese individuals (Age 24.93±6.39; BMI 33.94±3.08), 10 overweight individuals (Age 24.57±1.17; BMI 27.41±1.17), 20 healthy weight individuals (Age 23.9±5.61; BMI 21.94±1.94) | Resting state scans performed after overnight fasting (hunger) and following a standardized meal (satiety). | - During hunger, as compared to satiety, there was increased excitation of the ventromedial prefrontal cortex over the ventral striatum and hypothalamus.  - Increased BMI was associated with increased excitation of the anterior insula over the hypothalamus across the hunger and satiety conditions.  - The interaction of hunger and increased BMI yielded decreased intra-cortical excitation from the dorso-lateral to the ventromedial prefrontal cortex. |
| Burdette *et al*. (2020)/[101] | N=67 obese old adults (BMI 35.3±3.4) | Resting and food cue states | - The Power of Food Scale was most related to the visual cortex and sensorimotor processing areas during only the food cue state.  - During both the food cue and resting conditions, the Weight Efficacy Lifestyle Questionnaire was associated with the attention network and limbic circuitry. |
| Donofry *et al*. (2020)/[106] | N=122 overweight/obese adults (BMI 31.28±3.92) | Resting state scan and cue reactivity with high-caloric food, low-caloric food, non-food cues and Iowa gambling task | - BMI was associated with stronger FC during the presentation of high calorie food, but weaker FC at rest.  - Regions exhibiting BMI-related modulation of signal coherence in the presence of palatable food cues were largely located within the default mode network, while regions exhibiting BMI-related modulation of signal coherence at rest were located within the frontostriatal and DMN. |
| Li *et al*. (2021)/[111] | N=44 obese subjects (BMI 38.68±0.62), N=37 overweight subjects (BMI 28.10±0.20) and N=37 normal weight controls (BMI 20.78±0.29) | Resting state scan and cue reactivity with high- (HiCal) and low-caloric (LoCal) food cues | - Obese group had both greater basal activity and greater food cue-induced activation than overweight and normal weight groups.  - Overweight group had higher activity in the hippocampus/amygdala than the normal weight group, which was only significant during resting state.  - Mediation analysis showed that the relationship between BMI and hippocampus/amygdala response to HiCal food cues was mediated by their resting-state activity. |
| Park *et al.* (2018)/[114] | N=274 overweight people (BMI>25)  Participants were divided into abdominal (N=152, BMI 31.37±5.01) and non-abdominal obesity (N=122, BMI 29.84±4.40) groups using a waist–hip ratio | Resting-state fMRI and DTI | - Functional connectivity (degree centrality, DC) in FPN and ECN showed significant inter-group differences, and DC value in FPN was significantly associated with behaviors of eating disorders.  - Inter-group structural connectivity differences were observed in many brain regions. Among them, the anterior and posterior corona radiata and the cerebral peduncle yielded significant associations with behaviors of eating disorders.  - The highest association between the connectivity values and eating disorder scores was achieved by combining DC values of the cerebral peduncle, anterior corona radiata, posterior corona radiata (from structural connectivity), FPN (from static connectivity), and ECN (from dynamic connectivity) compared to the use of structural or functional connectivity only  - Multimodal imaging data is more effective than single-modal imaging data in responsible for behaviors of eating disorders in people with abdominal obesity |
| Ho *et al*. (2018)/[110] | N=17 seventeen patients with obesity (7 males and 10 females, BMI 37.99±5.40, age 31.82±8.75) | Diffusion tensor imaging, generalized q-sampling imaging, and resting-state functional magnetic resonance imaging | - Negative correlation between FA in the tapetum, RD in the corpus callosum and inhibitory control ability.  - Negative correlation AD in the posterior corona radiata and mental flexibility.  - Positive correlation between FA in the superior longitudinal fasciculus, RD, MD in the superior frontal blade and affective decision-making ability.  - Positive correlation between ISO in the posterior cingulate and inhibitory control; negative correlation between NQA in the posterior cingulate and inhibitory control  - Negative correlation between GFA in the posterior corona radiata, NQA in the precuneus and mental flexibility  - Positive correlation between the affective decision-making ability and GFA, NQA in the superior longitudinal fasciculus  - Positive correlation between the mfALFF in left precuneus, middle occipital gyrus and inhibitory control; negative correlation between the mfALFF in insula and inhibitory control  - Negative correlation between mental flexibility and mfALFF in the right vmPFC and angular gyrus  - Positive correlation between affective decision-making ability and mfALFF in the ACC, right precuneus and postcentral gyrus  - Negative correlations between inhibitory control and mReHo in the right dmPFC and putamen  - Positive correlation between mental flexibility and mReHo in the right OFC  Positive correlation between affective decision-making ability and mReHo in the right insula, whereas a negative correlation was found in the vmPFC |
| Park *et al*. (2020)/[55] | N=182 subjects (BMI 26.80±4.10) | Resting state scans and diffusion tensor imaging | - At a large-scale network-level, no networks showed significant interaction effects.  - At a finer node level, the orbitofrontal cortex showed interaction effects between periventricular WMH burden and degree of obesity.  - Higher functional connectivity was observed when the periventricular WMH burden and degree of obesity were both high. |
| **Structural MRI** | | | |
| Medic *et al*. (2016)/[83] | N=202 healthy subjects (Age range 18-50, BMI range 18.5-46.4) | T1 MRI | - Increasing BMI was not associated with global cortical changes.  - Reduction in cortical thickness in the left lateral occipital cortex (LOC) and right ventromedial prefrontal cortex (vmPFC) associated with increasing BMI.  - BMI-related thinning was separate from the age-related effects on thickness. |
| Saute *et al.* (2018)/[85] | N=44 teenagers (age 15-18): N=18 obese (BMI 31.11±3.21) and N=26 lean (BMI 21.38±1.70) | T1-weighted MRI | - No relationship of BMI or hepatorenal gradient with brain cortical dimensions  - Significant positive association between visceral fat ratio and cortical thickness throughout the brain  - Visceral fat, but not BMI, is correlated with cortical thickening in adolescence. |
| Beyer *et al*. (2019)/[82] | N=625 healthy adults (age 20-59, BMI 25.7±4.5) were enrolled in the “Health Study for the Leipzig Research Centre for Civilization Diseases” (LIFE-Adult) study | T1-weighted MRI | - Higher YFAS symptom score correlated with higher BMI, eating behavior traits, neuroticism, and stress.  - Higher BMI predicted significantly lower thickness of (pre)frontal, temporal and occipital cortex and increased volume of left nucleus accumbens.  - In a whole-brain analysis, YFAS symptom score was not associated with significant differences in cortical thickness or subcortical gray matter volumes  - A hypothesis-driven Bayes factor analysis suggested a small, additional contribution of YFAS symptom score to lower right lateral orbitofrontal cortex thickness over the effect of BMI.  - Symptoms of food addiction do not account for the major part of the structural brain differences. Yet, symptoms of food addiction might explain additional variance in orbitofrontal cortex |
| Ronan *et al*. (2020)/[84] | N=2700 children between the ages of 9 and 11 years | Structural images | - Increased BMI was associated with significantly reduced mean cortical thickness, as well as specific bilateral reduced cortical thickness in prefrontal cortical regions.  - Reduced thickness in the rostral medial and superior frontal cortex, the inferior frontal gyrus, and the lateral orbitofrontal cortex partially accounted for reductions in executive function. |
| Opel *et al*. (2021)/[81] | N=6420 subjects (BMI 25.97±4.97) | Structural images and genetic data | - The most pronounced effects were found for associations between obesity and lower temporo-frontal cortical thickness.  - A higher polygenic risk score for obesity significantly correlated with lower occipital surface area.  - A significant age-by-obesity interaction on cortical thickness emerged driven  by lower thickness in older participants. |
| Thapaliya *et al*. (2021)/[76] | N=22 lean adolescents with lean mothers (lean low-risk), N=25 lean adolescents with mothers with obesity/overweight (lean high-risk), N=36 adolescent with obesity/overweight | Structural images | - The lean high-risk compared with the lean low-risk group demonstrated lower gray and white matter volume and cortical thickness in the postcentral gyrus (somatosensory cortex), lower gray and white matter volume in the opercular cortex (taste cortex), lower gray matter volume and cortical thickness in the anterior cingulate cortex, and lower cortical thickness in the precuneus.  - Comparisons of the lean and obesity/overweight groups revealed further structural alterations in the postcentral gyrus, posterior cingulate gyrus, and middle temporal gyrus. |
| Mokhtari *et al*. (2016)/[70] | N=52 participants (Age range 60.70-79.80, BMI range 28.14-41.98) | T1-weighted anatomical images performed at baseline and 18-months after lifestyle interventions (3T MRI) | - Brain gray matter (GM) and white matter (WM) tissue volume at baseline was predictive of 18-months weight loss, the support vector machine resulted in an average classification accuracy of 72.62% based on gray matter and white matter volume.  - A receiver operating characteristic analysis indicated that classification performance was robust based on an area under the curve of 0.82. |
| Opel *et al*. (2017)/[72] | N=330 for the Münster Neuroimage Cohort (Age 39.2±11.3, BMI 24.5±3.9), 347 for the BiDirect study (Age 51.6±8.2, BMI 26.3±4.1) | T1-weighted 3D image | - Higher BMI and higher polygenic risk for obesity were significantly associated with medial prefrontal gray matter decrease.  - Prefrontal gray matter was further shown to significantly mediate the effect of polygenic risk for obesity on BMI in both samples. |
| Yokum *et al*. (2017)/[80] | N=34 body fat gain adolescents at baseline (BMI 21.5±2.0), 12 body fat stable adolescents (BMI 20.6±1.9), and 14 body fat loss subjects (BMI 20.3±1.4) | T1-weighted 3D image, 2- or 3-year follow-up | - Baseline global/regional GM/WM volume did not predict body fat gain over follow-up.  - Adolescents who gained body fat showed greater decreases in GM volume in the putamen compared with those who showed loss of body fat.  - Adolescents who gained body fat showed greater increases in WM volume in the anterior cingulate cortex compared with those who showed stability of or loss of body fat. |
| Wang *et al*. (2017)/[79] | N=31 obesity subjects (Age 39.58±1.93, BMI 34.38±0.69), and 49 normal weight controls (Age 29.55±1.41, BMI 21.87±0.29) | T1-weighted 3D image | - Reduced GM volumes were found in the frontal and limbic regions in the obese group compared to normal weight individuals.  - In the normal weight group, lack of perseverance was negatively correlated with GM volume in the anterior cingulate cortex, and negative urgency was negatively correlated with GM volume in the insula.  - In the obese group, sensation seeking was negatively correlated with GM volume in the left amygdala and right pallidum. |
| Sweat *et al*. (2017)/[75] | N=108 obese subjects (Age 19.60±1.54, BMI 35.57±4.97), 54 healthy weight controls (Age 19.39±1.52, BMI 21.45±1.87) | Structural images | - Groups differed on four measures of processing speed contained in four different cognitive tests, but not on executive function.  - Differences between the weight groups on the area of the anterior portion of the corpus callosum (CC), but not the overall CC. Only the Controlled Oral Word Association Test was significantly correlated with the area of the anterior portion of the CC.  - No differences were found between obese participants with or without metabolic syndrome (MetS) and none of the MetS factors contributed consistently to cognitive performance. |
| Nouwen *et al*. (2017)/[71] | N=15 adolescents with type 2 diabetes (T2DM), 21 obese adolescents, and 22 healthy weight controls | T1-weighted 3D image | - Adolescents with T2DM and obese adolescents had reduced gray matter volume in the right hippocampus, left putamen and caudate, bilateral amygdala and left thalamus compared to healthy weight controls.  - T2DM was also associated with significant regional changes in fractional anisotropy within the corpus callosum, fornix, left inferior fronto-occipital fasciculus, left uncinate, left internal and external capsule.  - Fractional anisotropy reductions within these tracts were explained by increased radial diffusivity, which may suggest demyelination of white matter tracts. Mean diffusivity and axial diffusivity did not differ between the groups. |
| Chao *et al*. (2018)/[103] | N=20 obese patients (BMI 37.66±5.07) and N=30 healthy controls (BMI 22.64±3.45) | Resting-state fMRI and T1-weighted imaging | - Obese patients showed increased gray matter volumes in the amygdala and putamen, decreased gray matter volumes in thalamus.  - Obesity group showed increased functional connectivity in the bilateral anterior cingulate cortex and decreased functional connectivity in the frontal gyrus of default mode network.  - Obesity group also exhibited altered ALFF and ReHo in the prefrontal cortex and precuneus  - Graph theoretical analysis revealed that obese patients showed a significant decrease in local segregation and a significant increase in global integration, suggesting a shift toward randomization in their functional networks |
| Perlaki *et al*. (2018)/[74] | N=51 Caucasian young subjects (32 females; age 13.8±1.9, range 10.2–16.5 years) | T1-weighted MRI | - The volumes of accumbens and amygdala showed significant positive correlations with age- and sex-standardized body mass index (zBMI), while their gray matter (GM) densities were inversely related to zBMI |
| Kakoschke *et al.* (2019)/[66] | N=127 individuals (BMI 25.69±5.15, age 24.79±9.60) | T1-weighted MRI | - The relationship between body fat and medial OFC volume was moderated by impulsivity  - Elevated impulsivity was associated with smaller amygdala and larger frontal pole volumes. |
| Singh *et al*. (2019)/[115] | N=42 depressed and overweight (BMI>85th%) youth aged 9 to 17 | Resting-state fMRI and T1-weighted MRI | - Youth with greater insulin resistance had higher levels of anhedonia and more food seeking behaviors, reduced hippocampal and ACC volumes, and greater levels of ACC and hippocampal dysconnectivity to fronto-limbic reward networks at rest  - For youth with high levels of insulin resistance, thinner ACC and smaller hippocampal volumes were associated with more severe depressive symptoms, whereas the opposite was true for youth with low levels of insulin resistance. |
| Parcet *et al*. (2020)/[73] | N=206 subjects (BMI 23.21±3.74) | Structural images | - Reward sensitivity and BMI were not significantly correlated.  - A relationship between BMI and reduced volume in the medial and lateral orbitofrontal cortex, and between reward sensitivity and lower striatum volume.  - The interaction between reward sensitivity and BMI was associated with individual differences in the hippocampal volume. |
| Thapaliya *et al*. (2021)/[76] | N=22 lean adolescents with lean mothers (lean low-risk) (BMI 20.4±2.3), N=25 lean adolescents with mothers with obesity/overweight (lean high-risk) (BMI 19.9±2.2) and N=36 adolescents with obesity/overweight (BMI 30.2±5.7) | Structural images | - The lean high-risk compared with the lean low-risk group demonstrated lower gray and white matter volume and cortical thickness in the postcentral gyrus (somatosensory cortex), lower gray and white matter volume in the opercular cortex (taste cortex), lower gray matter volume and cortical thickness in the anterior cingulate cortex, and lower cortical thickness in the precuneus.  - Comparisons of the lean and obesity/overweight groups revealed further structural alterations in the postcentral gyrus, posterior cingulate gyrus, and middle temporal gyrus. |
| Ludwig *et al*  (2021)/[67] | N=355 healthy subjects (Age 38.8; BMI 24.5) including 226 participants with normal weight and 98 overweight/obese participants | T1-weighted high-resolution anatomical images were acquired using a three-dimensional fast gradient echo sequence. | - Novelty seeking and BMI were associated positively with novelty seeking being a significant predictor of BMI.  - A significant negative association between novelty seeking and orbitofrontal cortex grey matter volume was found independent of BMI. |
| Tüngler *et al*  (2021)/[77] | N=502 participants (Age 55.3±12.0, BMI 27.4±4.2) with a mean follow-up-time of 4.9 years (Age 60.2±12.0, BMI 27.6±4.5) | Anatomic T1-weighted images were acquired | - Increased BMI values at baseline were associated with decreased brain parameters at follow-up, particularly pronounced for the orbitofrontal cortex and medial prefrontal cortex.  - The genetic predisposition for BMI had no effect on brain parameters at baseline or follow-up, |
| Pflanz *et al*  (2022)/[64] | N=15,634 subjects in the UK Biobank | The cerebral GM and WM phenotypes were derived from T1-weighted images and analyzed by an image-processing pipeline developed and run-on behalf of UK Biobank. Network measures were derived from the original diffusion MRI images. | - Central obesity was associated with decreased GM volume. Regional associations were found between central obesity and with specific GM subcortical nuclei (thalamus, caudate, pallidum, nucleus accumbens). |
| van Bloemendaal *et al*. (2016)/[95] | N=16 T2DM (Age 61.4±1.5, BMI 34.0±0.9), N=15 age- gender and BMI-matched normoglycemic obese subjects (BMI 32.6±0.8), and matched normoglycemic lean subjects (BMI 23.4±0.4) | Diffusion tensor imaging, and T1-weighted anatomical images | - Obese T2DM patients compared with lean subjects had lower axial diffusivity (in the right corticospinal tract, right inferior fronto-occipital tract, right superior longitudinal fasciculus and right forceps major) and reduced white matter volume (in the right inferior parietal lobe and the left external capsule region).  - In normoglycemic obese compared with lean subject’s axial diffusivity as well as white matter volume tended to be reduced, whereas there were no significant differences between normoglycemic obese subjects and T2DM patients.  - Only BMI was independently related to white matter integrity, and age, gender and BMI to white matter volume loss. |
| Kullmann *et al*. (2016)/[88] | N=16 lean (Age 26.68±3.68, BMI 22.43±1.61), 8 overweight (Age 26.12±1.95, BMI 28.13±1.38), and 9 obese participants (Age 26.88±4.45, BMI 33.16±3.16) | Diffusion tensor imaging | - White matter structures showed differences in MRI parameters consistent with reduced myelin, increased water and altered iron content with increasing BMI in the superior longitudinal fasciculus, anterior thalamic radiation, internal capsule and corpus callosum.  - BMI-related changes in DTI parameters revealed mainly alterations in mean and axial diffusivity with increasing BMI in the corticospinal tract, anterior thalamic radiation and superior longitudinal fasciculus. |
| Papageorgiou *et al*. (2017)/[90] | N=52 obese subjects (Age 52.0±15.7, BMI > 30), 96 overweight (Age 51.5±14.8, BMI range 25.0-29.9), and 120 normal weight subjects (Age 39.8±15.8, BMI range 18.5-24.9) | Diffusion tensor imaging | - Increased BMI was related with decreased fractional anisotropy in several white matter regions including the anterior and posterior thalamic radiation, the inferior fronto-occipital fasciculus, the inferior and superior longitudinal fasciculus, the corpus callosum (callosal body and forceps minor), the uncinate fasciculus, the internal capsule, the corticospinal tract and the cingulum (cingulate gyrus and hippocampus). |
| Zhang *et al*. (2018)/[96] | N=1255 participants (50% females, age 19-80, BMI 16.8-50.2) | Diffusion-weighted magnetic resonance imaging | - Higher BMI and higher waist-to-hip ratio (WHR) were associated with lower FA in multiple white matter tracts  - Higher BMI and higher WHR on executive functions through lower FA in fiber tracts connecting (pre)frontal, visual, and associative areas |
| Repple *et al*. (2018)/[91] | Two independent cohorts: A sample of n=369 healthy subjects from the Münster Neuroimaging Cohort (MNC), as well as a public available sample of n=1064 healthy subjects of the Humane Connectome Project (HCP) | Diffusion-weighted magnetic resonance imaging | - Higher BMI was significantly associated with decreased FA in the corpus callosum, bilateral posterior thalamic radiation, bilateral internal capsule and external capsule, bilateral inferior longitudinal fasciculus and inferior fronto-occipital fasciculus.  - No significant positive associations between BMI and FA occurred |
| Ottino-González *et al*. (2018)/[89] | N=31overweight-to-obese adults (BMI 30.75±4.86) and 21 lean controls (BMI 22.35±2.01) | Diffusion-weighted magnetic resonance imaging | - Overweight participants presented higher allostatic load indexes  - Higher allostatic load indexes correlated with lower fractional anisotropy in the inferior fronto-occipital fasciculi and the right anterior corona radiata, as well as with grey matter reductions in the left precentral gyrus, the left lateral occipital gyrus, and the right pars opercularis. |
| Estella *et al*. (2020)/[87] | N=17 obese females with binge eating disorder (BED) (BMI 36.07±0.8), N=13 obese females (OBC) (BMI 33.64±4.7) and N=17 normal-weight females (NWC) (BMI 22.50±2.0) | Diffusion tensor imaging | - The BED group (vs. NWC) had greater axial diffusion (AD) in the forceps minor, anterior thalamic radiation, superior and inferior longitudinal fasciculus.  - The BED (vs. OBC) group had greater fractional anisotropy in the forceps minor and greater AD in the superior longitudinal fasciculus, cingulate gyrus, and corpus callosum. |
| Spindler *et al*. (2020)/[93] | N=100 subjects (BMI range 18.44-41.76) | Diffusion tensor imaging | - Obesity was predicted by mean diffusivity of the anterior–superior cluster, suggesting altered inhibition of food intake. |
| Takeuchi *et al*. (2020)/[94] | N=435 healthy young  adults with mild obesity and without obesity (266 males: BMI 21.75±2.61, 169 females: BMI 21.16±2.15) | Diffusion tensor imaging | - The association between greater BMI and lower MD of the right globus pallidus and the right putamen. |
| **Multi-modalities MRI** | | | |
| Figley *et al*. (2016)/[107] | N=32 healthy subjects: 16 males (Age 28.7±9.7, BMI 26.2±4.4), and 16 females (Age 30.9±11.5, BMI 23.5±4.2) | Resting state scan, T1-weighted anatomical images, and diffusion-weighted images | - Higher BMI and body fat percent (BFP) were associated with widespread decreases in gray matter volume, white matter volume, and white matter microstructure.  - Higher BMI and BFP were associated with increased salience network (SN) functional connectivity and decreased white matter volumes throughout all three networks (i.e., the default mode network (DMN), executive control network (ECN), and SN). |
| Alarcón *et al*. (2016)/[99] | N=152 healthy adolescents ranging in BMI: 88 healthy weight (BMI 20.3±1.9), 46 overweight (BMI 24.2±1.6), 18 obese (BMI 30.9±5.4) | Spatial and verbal working memory task, diffusion-weighted images, and T1-weighted anatomical images | - BMI was inversely related to IQ and verbal and spatial working memory accuracy.  - No significant relationship between BMI and BOLD response for either verbal or spatial working memory.  - BMI was negatively correlated with FA in the left superior longitudinal fasciculus (SLF) and left inferior longitudinal fasciculus (ILF).  - ILF FA and IQ significantly mediated the relationship between BMI and verbal working memory performance, whereas SLF FA, but not IQ, significantly mediated the relationship between BMI and accuracy of both verbal and spatial working memory. |
| Steward *et al*. (2019)/[151] | N=24 obese women (BMI 42.67±7.11) and N=25 healthy controls (BMI 20.89±1.87) | Emotion regulation task and cognitive reappraisal task; Resting-state fMRI and Diffusion-weighted magnetic resonance imaging | - OB group presented reduced activation in the ventromedial prefrontal (vmPFC) cortex in comparison to the HC group when downregulating negative emotions, along with heightened activation in the extrastriate visual cortex  - Activation in vmPFC during cognitive reappraisal were negatively correlated with self-reported difficulties in emotion regulation  - OB patients exhibited decreased functional connectivity between the vmPFC and the temporal pole during rest  - OB showed decreased fractional white-matter track volume in the uncinate fasciculus |
| Byeon *et al*. (2019)/[102] | N=88 healthy weight (HW, BMI 22.5±1.64), N=88 overweight (OW, BMI 27.39±1.38), and N=88 obesity (OB, BMI 35.10±4.58) | DTI, resting-state functional magnetic resonance imaging (rs-fMRI), and T1-weighted data from enhanced Nathan Kline Institute-Rockland sample (NKI-RS) database | - Developed a spatially guided enhanced functional correlation tensor (s-eFCT) method  - The regional FA and MD values from the s-eFCT were used to predict BMI, and identified 26 FA features in regions of major fiber bundles, such as the corpus callosum (CC), fornix (FX), medial lemniscus (ML), internal capsule (IC), corona radiata (CR), fronto-occipital fasciculus (FO), cerebellar peduncle (CP), and cingulum including cingulate gyrus (CGC) and hippocampus (CGH) and 13 MD features in regions of the genu, body, and splenium of CC, FX, left ML, right cerebral peduncle, right anterior limb and left retro part of IC, both side of posterior thalamic radiation, left CGC, left CGH, and right uncinate fasciculus  - The correlation between real and predicted BMIs was 0.57 |
| Adise *et al*. (2021)/[98] | N=6852 children structural MRI data, 4856 children rsfMRI data, 4707 monetary incentive delay task data, 4000 stop signal task data and 4453 emotional n-back task data obtained from the ABCD study data version 2.0.1. | Structural magnetic resonance imaging, diffusion tensor imaging, resting state fMRI, and three task-based fMRI (monetary incentive delay task, stop signal task and emotional n-back task) scans. | - Widespread brain structure were associationed with BMI (e.g.,cortical thickness, surface area, subcortical volume, and DTI).  - Widespread rsfMRI inter- and intra-network correlations were related to BMI, as were regional activations on the working memory task.  - Pathological weight gain was predicted by structural features , but not by fMRI nor rsfMRI. |
| Chen *et al*. (2017)/[104] | N=36 female chronic dieters (Age range 18-23, BMI range 17.2-33.7) | Cue reactivity with food and control contrast images, diffusion tensor imaging | - A food cue reactivity task to localize the extent of the inferior frontal gyrus and orbitofrontal cortex involved in food-cue processing.  - A negative relationship between body fat percentage and white matter integrity within the identified tract. |
| Mestre *et al*. (2017)/[113] | N=12 obese children (Age 10.08±1.00, BMI 26.10±3.23), and 13 healthy weight controls (Age 10.38±1.26, BMI 17.71±1.90) | Taste paradigm, and T1-weighted image | - Children with OB, relative to HW, showed reduced left hippocampal volume, and greater response to taste in three clusters within the left hippocampus.  - Activation within the hippocampus was associated with eating in the absence of hunger and two subscales on a measure of eating behaviors (Food responsiveness, Food enjoyment). |
| de Groot *et al*. (2017)/[105] | N=23 obese adolescents (Age range 12-16), and 19 age-, sex-, and education- matched lean controls | T1-weighted anatomical images, and Stop Signal-and a Choice Delay Task | - Adolescents with obesity had greater volumes of the pallidum.  - In the group with obesity, greater pallidum volume was positively associated with the ability to delay reward in the Choice Delay Task. |
| Syan *et al*. (2019)/[116] | N=243 obese individuals (BMI 34.40±3.61) and N=469 lean individuals (control group, BMI 22.36±1.55) from HCP datasets | N-Back task using functional magnetic resonance imaging (fMRI) and T1-weight MRI | - Obese group exhibited significantly worse performance in terms of the National Institutes of Health Toolkit (NIH) 9-Hole Peg Board, Penn Working Memory Test, Delay Discounting, Penn Progressive Matrices, NIH Picture Vocabulary Test, Dimensional Change Card Sort Test and the in-scanner N-Back working memory test  - Obese group also exhibited significantly greater BOLD activation in N-Back task-negative regions, including the ventromedial prefrontal cortex, posterior cingulate, and right precentral gyrus  - Obese group exhibited significantly increased cortical thickness in the medial orbitofrontal cortex, rostral anterior cingulate cortex, inferior and superior parietal gyri, and decreased cortical thickness in temporal pole compared to controls |
| Gogniat *et al*. (2018)/[108] | N=88 older adults (BMI 26±4.34; Age 74.08±6.03) | Structural MRI and neuropsychological function assessed with the Repeatable Battery | - Significant positive association between BMI and total grey matter volume  - No association between BMI and neuropsychological functioning  - Moderately greater BMI in later life may modestly attenuate concomitant grey matter volume decline. |
| Gupta *et al*. (2017)/[109] | N=57 high BMI subjects (Age range 19.99-61, BMI range 25.00-43.59, 20 female), and 67 normal BMI (Age range 18-55, BMI range 18.19-24.80, 43 female) | Structural and diffusion tensor imaging | - In both males and females, individuals with high BMI (obese and overweight) had greater anatomical centrality (greater connectivity) of reward (putamen) and salience (anterior insula) network regions.  - Sex differences were observed both in individuals with normal and elevated BMI. In individuals with high BMI, females compared to males showed greater centrality in reward (amygdala, hippocampus and nucleus accumbens) and salience (anterior mid-cingulate cortex) regions, while males compared to females had greater centrality in reward (putamen) and sensorimotor (posterior insula) regions. |
| Augustijn *et al*. (2019)/[100] | N=18 obese children (BMI 31.64±4.35) and N=22 (BMI 16.85±1.15) healthy weight children | T1- and diffusion-weighted imaging scans | - Obese children showed significantly increased normalized clustering coefficient and small-worldness compared with healthy weigh controls.  - Obese children showed increased betweenness centrality, reduced clustering coefficient, and increased structural network strength, mainly in the motor cortex and reward network. |
| Medic *et al*. (2019)/[97] | Dataset1: N=52 subjects (age 25.44±5.27, BMI 27.46±6.11)  Dataset2: N=202 subjects (age 32.29±7.72, BMI 28.45±6.21)  Dataset3: N=897 subjects (age 28.82±3.68, BMI 26.65±5.29) from HCP | T1- and diffusion-weighted MRI | - Gaussian curvature of the white matter surface showed a significant, positive association with BMI across all three independent datasets  - Integrity of the white matter mediated the relationship between Gaussian curvature of the white matter surface and BMI |
| Thomas *et al*. (2019)/[117] | N1=338 adults (age 21-78, BMI 18-43)  Independent sample (N=236) | T1- and diffusion-weighted MRI | - Higher BMI predicted higher mean diffusivity (MD) within the hypothalamus.  - Hypothalamic volume was not associated with obesity.  - Larger hypothalamic volumes in the left compared to the right hemisphere |
| Rapuano *et al*. (2020)/[153] | N=5366 children (BMI 29.9±3.3) | Diffusion tensor imaging and restriction spectrum imaging, 1-year follow-up | - Cellular density in the NAc is related to individual differences in waist circumference at baseline and is predictive of increases in waist circumference after 1 y. |
| Dekkers *et al*. (2019)/[152] | N=12087 subjects (BMI 26.6±4.4, mean TBF in men was 24.4%±5.5, mean TBF in women was 35.5%±6.5) | Structural images and diffusion tensor imaging | - In men, TBF was negatively associated with all subcortical gray matter volumes (thalamus, caudate nucleus, putamen, globus pallidus, hippocampus, and nucleus accumbens) other than amygdala volume.  - In women, TBF was solely negatively associated with globus pallidus volume.  - In women and men, TBF was positively associated with global FA.  - TBF was negatively associated with global MD in women. |
