# Supplementary material for: Brain functional and structural magnetic resonance imaging of obesity and weight loss interventions
Source: Mol Psychiatry. 2023 Mar 14;28(4):1466–79. doi: 10.1038/s41380-023-02025-y (PMC10208984; doi:10.1038/s41380-023-02025-y)
Supplement: Supplementary file 2 — Supplementary Table 2 [file 41380_2023_2025_MOESM2_ESM.doc]

**Supplementary Table 2**. MRI studies related to weight-loss interventions.

| **Authors (year)/(ref #)** | **Study group** | **Imaging task** | **Main findings** |
| --- | --- | --- | --- |
| 1. **Treatment-Bariatric surgery** | | | |
| **Neural responsivity (fMRI)** | | | |
| Li *et al*. (2019)/[129] | N=22 obese patients (BMI 38.11±1.32) who received laparoscopic sleeve gastrectomy (LSG) and N=19 obese controls (BMI 35.27±1.01) without surgery | Cue-reactivity task with high- and low-calorie (LC) food pictures tested in obese patients before and one-month after LSG | - LSG significantly decreased fasting plasma concentrations of total ghrelin, leptin and insulin, craving for HC food, and brain activation in the right dorsolateral prefrontal cortex (DLPFC) in response to HC vs. LC food cues.  - LSG-induced reduction in DLPFC activation to food cues were positively correlated with reduction in ghrelin levels and reduction in craving ratings for food.  - LSG increased the connectivity between right DLPFC and the ventral anterior cingulate cortex (vACC), and changes in BMI were negatively correlated with changes in connectivity between the right DLPFC and vACC. |
| Baboumian *et al.* (2019)/[178] | Surgery groups  1. RYGB: N=16 obese patients, BMI 44.2±4  2. SG: N=9 obese patints, BMI 41.0±3  Control groups  1. low-calorie diet weight loss group (WL): N=14 obese patients, BMI 42.7±4  2. non-treatment group (NT): N=16, BMI 41.2±3 | Cue reactivity with high-energy dense (HED) vs. low-energy dense (LED) visual and auditory food cues  MRI scans performed before and approximately 4 months after surgery | - Surgery groups showed increased dorsolateral prefrontal cortex (DLPFC) and decreased parahippocampal/fusiform gyrus (PHG/fusiform) activation in response to HED vs. LED.  - DLPFC activation was significantly more increased in RYGB vs. SG.  Postprandial increases in GLP-1 concentrations (pre to postsurgery) correlated with postsurgical decreases in RYGB brain activity in the inferior temporal gyrus and the right middle occipital gyrus and with increases in the right medial prefrontal gyrus/paracingulate for HED > LED stimuli. |
| Holsen *et al*. (2018)/[177] | 18 obese patients with LSG surgery at baseline and 12-month post surgery. | Desire for palatable food regulation paradigm | - Brain activity in the nucleus accumbens (NAc), caudate, pallidum, and amygdala during desire for palatable food enhancement vs. regulation decreased from baseline to 12-months  - Dorsolateral and dorsomedial prefrontal cortex activity during desire for palatable food regulation (vs. enhancement) increased from baseline to 12-months  - Baseline activity in the NAc and hypothalamus during desire for palatable food enhancement was significantly predictive of %TWL at 12-months |
| Wang *et al*. (2016)/[176] | N=13 obese subjects (Age 46.5±9.3, BMI 43.1±4.4) eligible for gastric bypass surgery, and 7 non-obese healthy subjects (Age 51.7±7.8, BMI 27.0±2.2) served as controls | Taste stimulation task with sweet and salty solutions, 1-months and 1-year post-surgery follow-up | - After surgery brain activation in the reward center of the brain was significantly decreased in response to sweet solutions, but this effect was also seen in non-surgical controls.  - Surgical patients had significantly increased activation in the reward center to salty taste compared both to their preoperative scans and to healthy controls. |
| Zoon *et al*. (2018)/[179] | N=18 morbidly obese patients (15 female and 3 male; age 41±11 years; BMI 42±4 before RYGB; BMI 36±4 kg/m^2^ after RYGB) | Food-specific go/no-go task with pictures of high-energy and low-energy foods.  MRI scans performed before and after RYGB surgery (average 9.3 weeks, range 8-12 weeks, SD 1.2) | - RYGB increased activation of the right lateral prefrontal cortex (PFC), right medial PFC, dorsolateral PFC, right middle cingulate cortex and the right inferior frontal gyrus (involved in inhibitory control) in response inhibition to high-energy foods.  - RYGB decreased activation of left superior temporal pole, right parahippocampal gyrus and right hypothalamus (involved in metabolic control) in response inhibition to low-energy foods. |
| **Resting-state fMRI** | | | |
| Li *et al*. (2018)/[186] | N=22 obese participants (BMI 38.11±1.32/34.03±1.31) who received LSG and N=19 obese controls (BMI 35.27±1.01/35.14±1.04) without surgery tested at baseline and 1-month after LSG | Resting-state fMRI scans were performed before and one month after LSG | - Bariatric surgery significantly decreased lFCD in VMPFC, PCC/precuneus, and dACC/DMPFC and decreased gFCD in VMPFC, right DLPFC and right insula (pFWE < .05).  - lFCD decreases in VMPFC and PCC/precuneus correlated with reduction in BMI after surgery.  - VMPFC had stronger connectivity with left DLPFC and weaker connectivity with hippocampus/parahippocampus, and PCC/precuneus had stronger connectivity with right caudate and left DLPFC after surgery. |
| Zhang *et al.* (2019)/[127] | N=30 obese participants (BMI 38.32±0.80) who received laparoscopic sleeve gastrectomy (LSG) and N=26 obese controls (BMI 36.77±0.90) without surgery | Resting-state fMRI scans were performed before and one month after LSG | - One-month post-LSG there were significant decreases in appetite, body mass index (BMI), fasting plasma ghrelin and leptin levels, anxiety, and ALFF in HIPP and ALFF increases in posterior cingulate cortex (PCC).  - Decreases in HIPP ALFF correlated positively with decreases in fasting ghrelin and anxiety, and increases in PCC ALFF correlated positively with decreases in anxiety.  - Increased connectivity between HIPP and insula, and between PCC and dorsolateral prefrontal cortex (DLPFC) post-LSG. |
| Olivo *et al*. (2017)/[189] | N=16 severely obese women for Roux-en-Y gastric bypass surgery (Age 39±11, BMI 42.9±4.7), and 12 lean controls (Age 36±12, BMI 22.7±1.7) | Resting state scans, 1-month and 1-year follow-up | - Connectivity between regions involved in food-related saliency attribution and reward-driven eating behavior was stronger in pre-surgery patients compared to controls, but progressively weakened after follow-up.  - At one year, changes in networks related to cognitive control over eating and bodily perception also occurred.  - Connectivity between regions involved in emotional control and social cognition had a temporary reduction early after treatment but had increased again after one year of follow-up.  - Predict the BMI loss by pre-surgery connectivity in areas linked to emotional control and social interaction. |
| Li *et al*. (2018)/[187] | N=34 subjects with obesity and N= 34 age-and gender-matched normal-weight subjects, in which 17 subjects with obesity received sleeve gastrectomy. | Resting-state fMRI scans were performed and 4 months after surgery | - Preoperative subjects compared to controls had decreased resting-state activities in reward processing and cognitive control regions such as orbitofrontal cortex, middle frontal gyrus, superior frontal gyrus, and gyrus rectus, and these activities were negatively associated BMI  - Preoperative subjects had increased functional connectivity in above regions, such as increased FC between right superior orbitofrontal cortex and left thalamus, between right precentral cortex and bilateral superior frontal cortex, left, middle frontal cortex, and left medial superior frontal cortex, and these FCs were positively associated with BMI.  - After the surgery, most of these increased FCs were recovered. |
| Li *et al*. (2019)/[188] | N=38 obese patients (BM 40.0±6.5) and N=34 normal-weight controls (BMI 21.8±1.8). N=17 of 38 subjects underwent laparoscopic sleeve gastrectomy (LSG) (BMI 41.6±7.3) | Resting-state fMRI scans were performed before and four months after LSG | - Compared to normal-weight controls, pre-surgical subjects had increased functional connectivity (FC) between the reward region (putamen) and lateral hypothalamus (LH), and increased FC between somatosensory cortical area (insula) and medial hypothalamus (MH), as well as decreased FC between the cognitive control regions (prefrontal regions) and MH.  - Post-surgical FC between putamen and LH changed towards the patterns found in the control group.  - Changes in fasting glucose before and after the surgery were associated with the changes in FC between putamen and LH. |
| Cerit *et al*. (2019)/[184] | N= 14 obese patients (BMI 42.1±4.7) underwent laparoscopic sleeve gastrectomy (LSG) | Resting-state fMRI were performed before and 12-month after LSG | - LSG changes the magnitude (decreases) and directionality (positively correlated to anticorrelated) of functional connectivity (FC) within and between DMN, SN, and FPN nodes.  - Baseline FC of the nucleus accumbens (with insula) and hypothalamus (with precentral gyrus) predicted 12-month post-SG % total weight loss.  - Baseline FC of the hippocampus, FPN, and DMN nodes predicted improvement in cognitive control of eating behavior 12-months post-SG. |
| Gu *et al*. (2020)/[180] | N=24 obese patients (BMI 40.24±1.01) approved for LSG bariatric surgery and N=16 normal-weight patients with gastric cancer approved for gastric cancer surgery (GCS) (BMI 20.91±0.46) | Resting state scans performed before and 1 month after surgery | - Both LSG and GCS groups showed increased activities in the posterior cingulate cortex (PCC) and supplementary motor area (SMA) as well as the decreased RSFC of PCC-DMPFC and SMA-DLPFC.  - There were decreased resting-state activity of hippocampus and putamen in LSG group and increases in GCS group.  - In LSG group, resting-state activities of hippocampus and putamen were correlated with craving for high-caloric food and body mass index after surgery, respectively. |
| Heinrichs *et al*.  (2021)/[185] | N=33 obese patients (BMI 46.43±5.78) approved for RYGB (N=15), LSG (N=12) or gastric banding (N=1), and 15 obese waiting-list control patients (BMI 44.12±5.12) | Resting-state fMRI data at baseline, after 6 and 12 months. | - Bariatric surgery compared to waiting did not significantly affect functional connectivity of the reward network and the default mode network, neither whole-brain nor within-network.  - Surgery related BMI decrease and higher average head motion resulted in significantly stronger connectivity of the reward network with medial posterior frontal regions. |
| Wang *et al* (2022)/[183] | N=25 obese participants approved for LSG bariatric surgery (BMI 38.03±0.86) and 25 normal-weight controls (BMI 21.20 ± 0.42) | RS-fMRI images were acquired in normal weight subjects and obese subjects before LSG, 1- and 12-month post-surgery. | - LSG significantly increased local functional connectivity density in the mediodorsal thalamic nucleus (MD) and in the habenula (Hb) at 12-month post-surgery compared with baseline/1-month post-surgery, whereas LSG decreased local functional connectivity density in the posterior cingulate cortex/precuneus at 1- and 12-month post-surgery.  - The mediodorsal thalamic nucleus had stronger connectivity with precuneus and habenula at 12-month post-surgery, and the increased functional connectivity between precuneus and habenula correlated with decreases in hunger and BMI, respectively. |
| Wiemerslage *et al*.  (2017)/[181] | N=11 obese female for RYGB (Age 42±10, BMI 40.8 ± 4.0) | Resting state scans, in both a pre- and post-prandial state at two time points: four weeks before, and four weeks after bariatric surgery | - Bariatric surgery decreased resting-state activity in the claustrum, precentral gyrus, STG, putamen, insula, thalamus, and middle frontal gyrus.  - Increased activity in the cerebellum, superior frontal gyrus. |
| Zeighami *et al*  (2021)/[182] | N=57 participants with severe obesity (BMI 43.1±4.3 kg/m^2^) who underwent LSG, or RYGB. | RS-fMRI and T1-weighted anatomical images were performed prior to bariatric surgery and at follow-up visits of 4 months (N = 36), 12 months (N = 29), and 24 months (N = 14) after surgery | - There was a global increase in the fALFF signal with greater increase within dorsolateral prefrontal cortex, precuneus, inferior temporal gyrus, and visual cortex.  - The increase in neural activity was significantly related to post-surgery weight loss and improvement in cardiometabolic variables, such as blood pressure. |
| Zhang *et al*. (2021)/[190] | N=37 obese subjects (BMI 38.69±0.67) for LSG | Resting state scans performed before LSG surgery. | - Resting-state functional connectivity at baseline can be used to identify those obese patients with optimal weight loss after LSG with classification accuracy of 83.78%.  - Connectivity patterns contributing to the prediction consisted of complex multivariate network components in brain networks associated with salience, reward, self-referential, and cognitive processing.  - The connection strength between frontal and parietal cortices was stronger in the optimal versus the suboptimal weight loss group. |
| **Structural MRI** | | | |
| Liu *et al*. (2019)/[192] | N=22 obese participants who received laparoscopic sleeve gastrectomy (LSG) and N=21 obese controls without surgery | T1-weighted MRI scans were performed before and one month after LSG | - LSG significantly reduces the fasting plasma ghrelin, insulin, and leptin levels  - LSG significantly decreased the cortical thickness in precuneus and that was associated with decreases in BMI  - LSG increased cortical thickness in middle (MFG) and superior frontal gyri (SFG), superior temporal gyrus (STG), insula and ventral anterior cingulate cortex (vACC); and in cortical volume in left postcentral gyrus (PostCen) and vACC  - Changes in cortical thickness in the SFG were associated with decreases in BMI. |
| Bohon *et al*. (2018)/[194] | N=5 female obese patients | T1-weighted MRI scans were performed before and six-month after laparoscopic Roux-en-Y gastric bypass (RYGB) | - Increases in cerebral cortical thickness in patients with greater excess weight loss |
| Zhang *et al*. (2016)/[131] | N=15 obese subjects for laparoscopic sleeve gastrectomy surgery (BMI 38.10±1.50), and 18 age-, gender-matched normal weight controls (BMI 21.60±0.70) | T1-weighted images, and diffusion tensor imaging, 1-months follow LSG | - LSG increased FA in the anterior corona radiate, body CC, genu CC, fornix and sagittal stratum (SS).  - LSG increases GM density in the IFG, SFG, rACC, DMPFC, ITG, MTG, FFA and postcentral gyrus.  - Bariatric surgery generated partial neuroplastic structural recovery in the obese group, but the differences had relative less strength and smaller volume. |
| Tuulari *et al*.  (2016)/[130] | N=47 morbidly obese subjects (BMI 2.2±4.0) eligible for bariatric surgery, and 29 non-obese subjects (BMI 23.2±2.8) served as controls | T1-weighted anatomical images, 6-months follow BS | - Bariatric surgery results in global increase in WM volume.  - Bariatric surgery increase GM density in the occipital and inferior temporal regions.  - Decreases in plasma triglycerides, LDL cholesterol, and HbA1c after weight loss were associated positively with increased brain densities. |
| Bohon *et al*. (2019)/[195] | N=47 patients with severe obesity who participated in no treatment (N=16), behavioral weight loss (14), or bariatric surgery (17) | T1-weighted MRI scans were conducted pre-treatment and approximately four months after BS. | - No significant change in cortical thickness over time  - Significant increase in left hemisphere gray matter and white matter volumes over time  - At baseline and follow-up, there was no relationship between cortical thickness or brain volumes and BMI. |
| Wang *et al*. (2020)/[191] | N= 30 obese participants (BMI 39.15±0.80) who received laparoscopic sleeve gastrectomy (LSG) | T1-weighted MRI scans were performed at pre-LSG, 1-/3-month-post-LSG | - Increased GM volumes in caudate, INS and PCC, and increased WM volumes in ACC, PCC and PHIPP at 1-/3-month-post-LSG compared to pre-LSG.  - Increases in GM volumes were in caudate, PCC and IFG, and in WM volumes in ACC, PCC and PHIPP at 3-month relative to 1-month-post-LSG.  - GM volumes in INS and PCC showed a positive correlation at 1-month and 3-month-post-LSG.  - GM volume in INS and PCC were positively correlated with RSFC of INS-PCC and PCC-INS at 1 and 3-month-post-LSG. |
| Michaud *et al*. (2020)/[196] | N=29 obese patients (BMI 44.1±4.6) approved for LSG bariatric surgery and N=46 normal-weight controls (BMI 22.2±1.3) | Structural images at baseline, 4 and 12 months after LSG | - Increased WM density in the cerebellum, brain stem, cerebellar peduncle, cingulum, corpus callosum and corona radiata after surgery.  - Significant increases in GM density were observed 4 months after SG compared to baseline in the bilateral occipital cortex, temporal cortex, postcentral gyrus, cerebellum, hippocampus and insula as well as right fusiform gyrus, right parahippocampal gyrus, right lingula gyrus and right amygdala.  - These GM and WM increases were more pronounced and widespread after 12 months and were significantly associated with postoperative weight loss and the improvement of metabolic alterations. |
| Wang *et al.* (2022)/[154] | N=30 obese participants approved for LSG bariatric surgery (BMI 38.06±0.82) and 30 normal-weight controls (BMI 21.42±0.46) | Diffusion weighted imaging was acquired before LSG and 12-month post-LSG. | - LSG increased structural connectivity between the habenular and homeostatic/hedonic regions including hypothalamus, bilateral superior frontal gyri, left amygdala, and orbitofrontal cortex.  - Increased structural connectivity of habenular-hypothalamus correlated with reduced depression and DEBQ-external eating.  - Increased structural connectivity of habenular-left amygdala correlated with reduced DEBQ-emotional eating. |
| Li *et al.* (2022)/[193] | N=25 obese participants approved for LSG bariatric surgery (BMI 37.88±0.88) and 12-month after LSG (BMI 26.80±0.91) | Diffusion weighted imaging was  acquired before LSG and 12-  month post-LSG. | - Increases in fractional anisotropy and axial diffusivity between the right insula and anterior cingulate cortex, and higher fractional anisotropy of left insula-putamen, left insula-caudate and anterior cingulate cortex-right posterior cingulate cortex/precuneus at PostLSG_12_ compared with PreLSG.  - Negative correlations between axial diffusivity of right insula-anterior cingulate cortex and body mass index, and fractional anisotropy of right insula-anterior cingulate cortex with scores on external eating at PostLSG_12_.  - Decrease in resting-state functional connectivity between left insula and left caudate |
| **Multi-modalities MRI** | | | |
| Rullmann *et al*. (2018)/[197] | N=27 patients (BMI 47.8±5.5) undergoing RYGB and 14 non-obese matched controls (BMI 24.7±3.4) | Resting-state, T1-weighted and DTI MRI scans performed before and six-month, one-year after RYGB | - Patients presented widespread changes in white matter density (WMD) as well as gray matter density (GMD) in the cerebral cortex of all lobes, subcortical structures, the brainstem as well as the cerebellum.  - No changes in white matter diffusivity throughout the brain.  - GMD and WMD changes were associated with elevated ReHo.  - RYGB induces widespread plastic changes in brain structure that concurrently homogenizes the functional profile of the cortex, subcortical regions matter structures. |
| Hu *et al*. (2020)/[198] | N=28 obese patients (BMI 39.3±0.9) approved for LSG bariatric surgery and N=22 obese controls without surgery (BMI 36.9±1.0) | Diffusion tensor imaging (3T MRI) and cue reactivity with high-calorie food, low-calorie food pre-surgery and 1-month post-surgery. | - LSG significantly decreased right dorsolateral prefrontal cortex (DLPFC) activation to high-calorie food versus low-calorie food cues.  - LSG increased FC and SC between DLPFC and anterior cingulate cortex (ACC).  - Increases in SC and FC between DLPFC and ACC were associated with greater reductions in BMI, and SC changes were positively correlated with FC changes. |
| Hu *et al*. (2021)/[128] | N=25 obese subjects (BMI 38.78±0.75) approved for LSG bariatric surgery and 30 normal weight subjects (BMI 21.31±0.47). | Diffusion-weighted images and functional images with Food-Cue-Reactivity Task (high-calorie and low-calorie food) were acquired in normal weight subjects and obese subjects before LSG and 1- and 6-month post-surgery. | - LSG increased functional connectivity between the right dorsolateral prefrontal cortex and the pregenual anterior cingulate cortex (DLPFC-pgACC) and increased structural connectivity between DLPFC and ACC at 1 month and 6-month after LSG.  - Reduction in craving for high-calorie food cues correlated negatively with increased functional connectivity of DLPFC-pgACC at 6-month after LSG.  - Reduction in BMI correlated negatively with increased structural connectivity of DLPFC-ACC at 1 month and 6-month after LSG.  - Structural connectivity of DLPFC-ACC mediated the relationship between lower ghrelin levels and greater cognitive control. |
| 1. **Treatment-Dietary and Lifestyle Intervention** | | | |
| Drummen *et al*. (2018)/[201] | N=12 participants in the moderate protein (MP) dietary guidelines group, and 15 participants in the high protein (HP) dietary guidelines group | Food cue reactivity with high-calorie food, low-calorie food, and non-food items, 2-year follow-up | - Brain reactivity, BMI, HOMA-IR and protein intake did not change differently between the groups during the intervention.  - In the whole group, protein intake during weight maintenance was negatively related to changes in high calorie images>low calorie images (H > L) brain activation in the superior/middle frontal gyrus and the inferior temporal gyrus H > L brain activation was positively associated with changes in body weight and body-fat percentage and inversely associated with changes in dietary restraint in multiple reward, gustatory processing regions. |
| Simon *et al*. (2018)[40]/ | N=33 overweight women (BMI 27-40) at least 6 months after the completion of a diet, N=17 women were able to maintain weight loss (BMI 29.1±5.2), N=16 showed weight regain (BMI 33.4±6.5). | Participants performed incentive delay tasks with monetary or food rewards under hunger condition and satiety condition. | - Only participants with successful weight loss maintenance showed a satiety-induced attenuation of brain activation in VS during the expectation and in medial OFC during the receipt of a food-related reward.  - Satiation-induced decreases in active ghrelin levels were related to satiation-induced decreases in the VS during the expectation of food-related reward in the Maintain group.  - Attenuated influence of satiety signaling on the neural processing of food-related reward contributes to unsuccessful weight loss maintenance. |
| Hermann *et al.* (2019)/[200] | N=29 obese adults (26 women, age 47.59±12.64, BMI 36.88±5.50). | - Food stimuli with neutral objects, high- and low-calorie foods images.  - Participants were scheduled for two separate fMRI imaging sessions, one before starting the six-month diet weight loss intervention (baseline, Session 1) and another at the end of the first month of the six-month weight loss intervention (Session 2). | - Significant positive correlation between BMI change measured after six months and early alterations of fMRI food cue reactivity in the striatum, including the bilateral putamen, right pallidum, and left caudate.  - Higher fMRI responses in the dorsal and ventral regions of the bilateral putamen, as well as in the right pallidum and caudate at the first month of the intervention (Session 2) were associated with less weight loss  - No significant association between fMRI responses to high- vs. low-calorie food images measured at baseline (Session 1) and BMI change after the six-month intervention |
| Honea *et al*. (2016)/[65] | N=72 dieters with obesity (DwO, Age 38.9±8.2, BMI 35.6±3.6), 22 healthy weight controls (HW, Age 36.8±10.9, BMI 21.6±1.6) | T1-weighted anatomical images performed at baseline and 3-months after lifestyle interventions | - The percent weight loss (%WL) was positively correlated with baseline gray matter volume (GMV) in right parahippocampal and orbitofrontal gyri in DwO.  - Successful dieters showed greater GMV loss in the left precentral gyrus and the insula compared with unsuccessful dieters.  - A negative correlation was found between %WL and GMV change from baseline in the left prefrontal regions. |
| Stillman *et al*. (2021)/[202] | Diet-only intervention (N=42, BMI 32.26±3.6), Diet + moderate exercise (N=25, BMI 32.16 ±4.4), and Diet + high exercise (N=40, BMI 32.22±4.1) | T1-weighted anatomical image, perfusion-weighted images were performed at baseline and after 12 months intervention | - A 12-month diet and exercise program resulting in 10% weight loss increased cerebral blood flow. These effects were widespread and extended throughout frontal, parietal, and subcortical regions.  - There was some regional specificity of effects for both diet-only and diet combined with exercise. |
| Legget *et al*. (2016)/[203] | N=11 overweight/obese individuals (Age 38.2±3.2, BMI 33.6±1.4) | Resting state scans performed at baseline, and 6-months after exercise program | - Following the intervention, between-network connectivity (BNC) in the posterior cingulate cortex (PCC) was significantly reduced.  - Significant reductions in both outgoing causal flow from the PCC to a number of networks (language network, visual network, sensorimotor network, left executive control network, basal ganglia network, posterior default mode network), in addition to reductions in ingoing causal flow to the PCC from a number of networks (ventral default mode network, language network, sensorimotor network, basal ganglia network) following exercise.  - Change in BNC was related to changes in aerobic fitness level and perceived hunger. |
| Mokhtari *et al*. (2018)/[204] | Overweight and obese older adults (age:65-79, 28<BMI<42) | Resting-state fMRI scan before an 18-month lifestyle weight loss intervention. | - The predictive model combining machine learning and functional brain networks for weight loss outcome achieve an accuracy exceeded 95%.  - Connectivity patterns that contributed to the prediction consisted of complex multivariate network that are associated behavior emergence, self-regulation, body awareness, and the sensory features of food. |
| Esteban-Cornejo *et al*.  (2021)/[121] | N=99 children with overweight/obesity (10.0±1.1 years; 60 boys and 39 girls; BMI 26.7±3.7) | Resting-state fMRI data | - Cardiorespiratory fitness was independently associated with greater hippocampal connectivity between anterior hippocampus and frontal regions (β ranging from 0.423 to 0.424, p< 0.001). 发现海马连接的增强，维持长久减重  - Motor fitness was independently associated with diminished hippocampal connectivity between posterior hippocampus and frontal regions (β ranging from -0.583 to -0.694, p< 0.001). |
| Levakov *et al*. (2021)/[205] | Physical activity (PA) intervention (N=29, Weight 90.3±12.5), PA+ Mediterranean (MED) intervention (N=29, Weight 91.25±10.5), and PA + MED + polyphenols intervention (N=34, Weight 90.6±10.9) | Resting state scans performed at baseline and after 6 months of a lifestyle intervention | - A robust subnetwork composed mainly of sensory and motor cortical regions, whose edges correlated with future weight loss. This effect was found regardless of intervention group.  - The engagement of senso-motor regions in this subnetwork is consistent with the over-sensitivity to food cues theory of weight regulation.  - Tested an additional hypothesis regarding the role of brain-gastric interaction in this subnetwork, and found a significant spatial overlap with the subnetwork reported in the present study.  - Power in the gastric basal electric frequency within the subnetwork negatively correlated with future weight loss. |
| Espeland *et al*. (2016)/[207] | N=319 participants receive 10 years of lifestyle intervention: 155 with a diabetes support and education, and 164 with an intensive lifestyle intervention | T1-weighted anatomical images, 10-year follow-up | - Total brain and hippocampus volumes were similar between intervention groups.  - The mean white matter hyperintensity volume was 28% lower among lifestyle intervention participants compared with those receiving diabetes support and education. The mean ventricle volume was 9% lower.  - Assignment to lifestyle intervention was not associated with consistent differences in cognitive function compared with diabetes support and education. |
| Rodriguez-Ayllon *et al*. (2020)/[206] | N=103 subjects (BMI 26.72±3.62) | Diffusion tensor imaging | - Self-reported total physical activity (PA) was positively associated with Global FA while watching television was negatively associated with Global FA.  - No association was found between PA and sedentary behavior and FA and MD within individual tracts. |
| 1. **Treatment-Pharmacology** | | | |
| Ten *et al*. (2016)/[208] | N=20 obese patients with type 2 diabetes, treatment with insulin and liraglutide | Food cue reactivity task performed at baseline and after 12-week treatment | - After 12 weeks, the decrease in HbA1c was larger with liraglutide versus insulin glargine. Body weight decreased during liraglutide versus insulin glargine.  - After 10 days, patients treated with liraglutide, compared with insulin glargine, showed decreased responses to food pictures in insula and putamen.  - Liraglutide enhanced the satiating effect of meal intake on responses in putamen and amygdala. Differences between liraglutide and insulin glargine were not observed after 12 weeks. |
| Farr *et al*. (2016)/[212] | N=21 obese patients with type 2 diabetes, treatment with liraglutide and placebo | Food cue reactivity task performed at baseline and after 17 days treatment | - Immunohistochemical analysis revealed the presence of GLP-1 receptors on neurons in the human hypothalamus, medulla and parietal cortex.  - Liraglutide decreased activation of the parietal cortex in response to highly desirable (vs less desirable) food images; decreased activation in the insula and putamen, areas involved in the reward system.  - Increased ratings of hunger and appetite correlated with increased brain activation in response to highly desirable food cues while on liraglutide, while ratings of nausea correlated with decreased brain activation. |
| Farr *et al*. (2019)/[213] | N=28 obese patients were randomized 1:1 to receive liraglutide or placebo | Food cue reactivity task | - While using liraglutide, patients lost more weight, had decreased fasting glucose and showed improved cholesterol levels.  - In an uncontrolled analysis, brain activation in response to food images was not altered by liraglutide vs placebo.  - When controlled for BMI/weight, liraglutide increased activation of the right orbitofrontal cortex (OFC) in response to food cues. |
| van Ruiten *et al*. (2022)/[222] | N=10 obese patients with type 2 diabetes treatment with insulin glargine, and N=10 treatment with liraglutide | Food cue reactivity with high-calorie food, low-calorie food, and non-food items, which performed after treatment (10 days, 12 weeks) | - After 10 days, higher emotional eating scores were associated with less pronounced GLP-1RA induced reductions in brain responses to food pictures in the amygdala, insula and caudate nucleus; Baseline external eating scores were associated with less pronounced GLP-1RA induced reductions in brain responses to food pictures in the insula, amygdala and orbitofrontal cortex.  - Higher emotional eating scores tended to be associated with less pronounced GLP-1RA increases in brain responses to chocolate milk receipt in the caudate nucleus and insula.  - After 12 weeks, there were no significant associations between emotional eating scores and liraglutide-induced changes in brain responses to food cues; Baseline restraint eating scores were associated with more GLP-1RA induced reductions in brain responses to food pictures in the insula and caudate nucleus, and with more GLP-1RA induced reductions in brain responses to the anticipation of chocolate milk in the caudate nucleus. |
| Heni *et al*. (2017)/[220] | N=21 healthy men volunteers received intranasal insulin or placebo | Pulsed arterial spin labeling measurement, before and thirty minutes after administration of the spray | - Glucose uptake into tissue tended to be higher after nasal insulin application. No such effects were detected in overweight participants.  - The increase in glucose infusion rates was associated with regional brain insulin action in hypothalamus and striatum.  - Suppression of endogenous glucose production by circulating insulin was more pronounced after administration of nasal insulin than after placebo. |
| Kullmann *et al*. (2017)/[219] | N=25 healthy lean, 10 overweight, and 12 obese adult participants received intranasal insulin or placebo | Resting state scans performed at before and thirty minutes after insulin/placebo spray | - Insulin compared to placebo administration resulted in increased functional connectivity between the prefrontal regions of the default-mode network and the hippocampus as well as the hypothalamus.  - The intranasal insulin induced hippocampal functional connectivity increase served as a mediator, suppressing the relationship between visceral adipose tissue and hunger.  - The insulin-induced hypothalamic functional connectivity change showed a significant positive interaction with peripheral insulin sensitivity. |
| Kullmann *et al*. (2018)/[123] | N=9 healthy men received placebo or 40 U, 80 U, and 160 U insulin spray in randomized order | Resting state scan performed before and after nasal spray application. | - Nasal insulin administration dose-dependently modulated regional brain activity and the normalized high-frequency component of the heart rate variability.  - Nasal insulin dose-dependently modulated regional brain activity with the strongest effects after 160 U. |
| Striepens *et al*. (2016)/[124] | N=31 healthy adult females (BMI 22.26±3.03) who received 24 IU of intranasal neuropeptide oxytocin (OXT) or placebo | Food cue reactivity task | - The neuropeptide oxytocin (OXT) specifically reduced food craving in the cognitive control condition. These findings were paralleled by an increase of activity in the middle and superior frontal gyrus, precuneus, and cingulate cortex under OXT.  - Interestingly, the behavioral OXT effect correlated with the OXT-induced changes in the prefrontal cortex and precuneus. |
| Kerem *et al*. (2020)/[221] | N=10 men with overweight or obesity (BMI 28.9±0.8) were randomized 1:1 to one of two drug orders (i.e., oxytocin-placebo or placebo-oxytocin) | Food cue reactivity task | - Following oxytocin (OXT) administration, participants exhibited significantly attenuated functional connectivity between the ventral tegmental area (VTA) and the insula, oral somatosensory cortex, amygdala, hippocampus, operculum, and middle temporal gyrus in response to viewing high-calorie foods.  - There was no difference in functional connectivity between VTA and these brain areas when comparing OXT and placebo for low-calorie food, nonfood, and fixation images. |
| van Ruiten *et al*. (2022)/[222] | N=16 obese patients with type 2 diabetes treatment with dapagliflozin, 17 treatment with exenatide, 16 treatment with dapagliflozin + exenatide, and 16 treatment with placebo | Food cue reactivity with high-calorie food, low-calorie food, and non-food items, which performed after treatment (10 days, 16 weeks) | - Dapagliflozin versus placebo decreased activity in response to low-calorie food pictures, in the caudate nucleus, insula, and amygdala after 10 days, and in the insula after 16 weeks.  - Exenatide versus placebo increased activation in the putamen in response to low-calorie food pictures after 10 days, but not after 16 weeks.  - Dapagliflozin plus exenatide versus placebo had no effect on brain responses, but after 10 days dapagliflozin plus exenatide versus dapagliflozin increased activity in the insula and amygdala in response to low-calorie food pictures. |
| Wang *et al*. (2018)/[122] | N=36 obese women treatment with the combination of sustained release (SR) naltrexone and SR bupropion (NB32, N=16) or with placebo (N=20) | Resting state scans performed at baseline and after 4-week treatment with NB32 or placebo. | - After NB32 treatment, the group showed lower local and global FCD than the placebo group in the right superior parietal gyrus and lower local FCD in the left middle frontal gyrus.  - Right superior parietal gyrus showed higher positive FC with the dorsal anterior cingulate gyrus, bilateral insula, and left superior parietal gyrus and stronger negative FC with right inferior frontal gyrus and right superior parietal gyrus for the NB32 than the placebo group.  - The NB32 group showed a significant correlation between local FCD change after treatment in left middle frontal gyrus and craving control scores. |
| 1. **Treatment-Neuromodulation** | | | |
| Kim *et al*. (2019)/[134] | N=36 subjects with obesity (54.1±11.0 years, BMI 30.2±3.5, 77.8 % females). N=20 in the real stimulation (TMS group, and N=16 in the sham stimulation group | Resting-state fMRI.  A total of 8 sessions of high-frequency rTMS targeting the left DLPFC were provided over a period of 4 weeks. | - Participants in the real stimulation group showed significantly greater weight loss from baseline following the 8 sessions of rTMS.  - rTMS increased the betweenness centrality values within the right frontoparietal network. |
| Devoto *et al*. (2021)/[223] | N=9 treated with high-frequency stimulation (realTMS), N=8 treated with sham (shamTMS) | Resting-state fMRI were acquired at baseline and 5-week later. | - Increase in degree centrality for the realTMS group in the MOFC and a decrease in the occipital pole. |
| Fatakdawala *et al*. (2021)/[224] | N=43 randomly assigned to 1) iTBS targeting left DLPFC, 2) bilateral DMPFC, 3) sham. | Intermittent theta-burst stimulation (iTBS) targeting the DLPFC and DMPFC, two cognitive task (delay discounting (DD), Flaner), a taste test. | - fNIRS revealed increases in MPFC activity when stimulate DMPFC during DD task.  - Neural efficiency effect was observed in the DLPFC stimulation during Flanker. |
| Ghobadi-Azbari *et al*. (2022)/[133] | N=64 overweigh/obesity, | One session active or sham tDCS over the DLPFC (anode F4 and cathode F3, 2 mA intensity for 20 min). | - Changes in response to food cue in the ventral striatum after a single-session  - Changes in food craving evaluated by the Food Craving Questionaire-State. |
| Kohl *et al*. (2019)/[132] | N=38 subjects with overweight/obesity (BMI 25-40 kg/m^2^). N=17 neurofeedback training with left DLPFC, and N=21 neurofeedback training with visual cortex. | Training session on a single day including 3 training runs of 6 trials of up-regulation and passive viewing for 4 weeks. Food appraisal and snack intake were assessed before and after training. | - Neurofeedback training increased functional connectivity between DLPFC and VMPFC which is involved with processing food value during up-regulation compared to passive viewing.  - Both groups rated pictures of high- but not low-calorie foods as less palatable and chose them less frequently. |
